# Back to the Roots: *Agrobacterium*-Specific Phages Show Potential to Disinfect Nutrient Solution from Hydroponic Greenhouses

**DOI:** 10.1128/aem.00215-23

**Published:** 2023-04-03

**Authors:** K. J. Fortuna, D. Holtappels, J. Venneman, S. Baeyen, M. Vallino, P. Verwilt, H. Rediers, B. De Coninck, M. Maes, J. Van Vaerenbergh, R. Lavigne, J. Wagemans

**Affiliations:** a Department of Biosystems, KU Leuven, Leuven, Belgium; b Leuven Plant Institute, KU Leuven, Leuven, Belgium; c Flanders Research Institute for Agriculture, Fisheries and Food (ILVO), Ghent, Belgium; d Institute for Sustainable Plant Protection, National Research Council of Italy, Turin, Italy; e Laboratory for Process Microbial Ecology & Bioinspirational Management (PME&BIM), Centre of Microbial and Plant Genetics (CMPG), Leuven, Belgium; f Ghent, Belgium; University of Nebraska-Lincoln

**Keywords:** bacteriophage biocontrol, hairy root disease, phage resistance, phage characterization, *Agrobacterium* biovar 1

## Abstract

*Agrobacterium* biovar 1 is a soilborne plant pathogen with the ability to colonize the irrigation system of greenhouses, causing hairy root disease (HRD). Currently, management focuses on using hydrogen peroxide to disinfect the nutrient solution, but due to the emergence of resistant strains, its efficacy and sustainability are questioned. Using a relevant collection of pathogenic *Agrobacterium* biovar 1 strains, OLIVR1 to 6, six phages specific to this pathogen and belonging to three different genera were isolated from *Agrobacterium* biovar 1-infected greenhouses. All phages were named OLIVR, referring to their location of isolation, Onze-Lieve-Vrouwe-Waver, and were characterized by whole-genome analysis, confirming their strictly lytic lifestyle. They remained stable under greenhouse-relevant conditions. To assess the efficacy of the phages, their ability to disinfect greenhouse nutrient solution inoculated with agrobacteria was tested. Each of the phages infected their host, but their ability to decrease the bacterial concentration differed. For instance, OLIVR1 reduced the bacterial concentration with 4 log units without phage resistance emerging. While OLIVR4 and OLIVR5 were also infectious in nutrient solution, they did not always decrease the bacterial load below the limit of detection, and phage resistance emerged. Finally, the mutations causing phage resistance by receptor modification were identified. For OLIVR4-resistant *Agrobacterium* isolates, but not for OLIVR5-resistant isolates, motility decreased. Together, these data show the potential of some of these phages as disinfectant of nutrient solution, and they might be a valuable tool to tackle HRD.

**IMPORTANCE** Hairy root disease, caused by rhizogenic *Agrobacterium* biovar 1 is a rapidly emerging bacterial disease worldwide. It affects tomatoes, cucumbers, eggplant, and bell pepper, causing high yield losses in hydroponic greenhouses. Recent findings suggest that the current management practices, mainly focusing on UV-C and hydrogen peroxide to disinfect contaminated water, have a questionable efficacy. Hence, we investigate the potential of phages as a biological means of preventing this disease. Using a diverse collection of *Agrobacterium* biovar 1, we isolated three different phage species that together infect 75% of the collection. Since these phages are strictly lytic, while remaining both stable and infectious under greenhouse-relevant conditions, they might be suitable candidates for biological control.

## INTRODUCTION

The global population has exceeded 8 billion people and is expected to reach 9.7 billion by 2050 (1). Feeding this growing population remains a challenge, partly due to extreme weather events and changing environmental conditions (e.g., changes in water quality and quantity) but also because of the global spread of plant pests and diseases (2, 3). These account for crop production losses of around 10 to 28% (4). Some of the most notorious diseases are caused by plant-pathogenic bacteria, mostly belonging to the *Xanthomonadaceae*, *Pseudomonadaceae*, and *Enterobacteriaceae*. Severe bacterial outbreaks are estimated to result in production losses of around 10% at the farm level (5–7).

Agrobacteria (family *Rhizobiaceae*) are considered one of the most important phytopathogenic bacteria, causing diseases in various production systems. Classification of this highly diverse group has been the subject of fierce debate over the years. Traditionally, strains belonging to *Agrobacterium* have been classified within three different biovars based on phenotypic and metabolic characteristics. Advances in sequencing technologies have shed new light on the genomic relationships of these bacteria, which largely correspond to the previously identified biovars. The biovar 1 strains, also known as the *Agrobacterium* species complex, currently consist of 15 described genomospecies (8–10). They are all characterized by the presence of a circular and linear chromosome. The biovar 2 strains are currently classified as Rhizobium rhizogenes, characterized by a primary chromosome and megaplasmid. The biovar 3 strains are currently classified as Allorhizobium vitis and possess two circular chromosomes (9). Alongside the genome, each of the biovars can also contain plasmids that determine the virulent nature of *Agrobacterium*. Depending on the disease caused, two plasmid types are distinguished: tumor-inducing plasmids (pTi) triggering typical crown galls, root galls, and root-plant galls (11) and root-inducing plasmids (pRi) causing extensive root proliferation (12).

In addition, the roots gain the ability to produce and secrete opines, which serve as a carbon and nitrogen source for *Agrobacterium* (13). Over the past decade, the impact of this disease on hydroponic cultivation systems has rapidly increased (14, 15), and currently, 45% of the Flemish tomato greenhouses are infested with this pathogen (16), resulting in average yield losses of around 10% (14). Several other countries, including Japan, Canada, Russia, New Zealand, and South Korea have reported a similar progression of the disease (14, 17, 18).

The most practiced strategy to control infections is prevention, using mainly hydrogen peroxide and UV-C. However, various *Agrobacterium* strains have developed high tolerance to hydrogen peroxide, limiting its efficacy (19). This is further exacerbated by the formation of bacterial biofilms in the greenhouse tubing system. Moreover, the use of hydrogen peroxide is preferentially avoided due to its off-target effect on other, potentially beneficial species. UV-C, on the other hand, has the disadvantage that it is local and cannot reach the biofilms in which these bacteria reside. This makes the efficacy of the current disease management practices questionable. As a consequence, the implementation of biological control agents, has been proposed as an alternative disease management strategy (18, 20, 21). In this regard, the use of bacteriophages to control bacterial plant diseases has received keen interest since the beginning of the 21st century, due to a better understanding of their biology combined with their ease of production, high specificity, and low environmental impact (22). A number successful trials have shown promising biocontrol effects against different genera, including *Acidovorax*, *Xanthomonas*, *Xylella*, Pseudomonas, *Pectobacterium*, *Ralstonia*, and *Erwinia* (23). For *Agrobacterium* specifically, the use of phages dates back to 1967, when Sonier and colleagues discovered *Agrobacterium* phage PB2, a phage that inhibited tumor formation in Nicotiana tabacum and Nicotiana glauca (24). Today, 13 *Agrobacterium*-specific phage genomes are accessible in public databases, and there has been some interest in using them as biocontrol organisms. For instance, Attai and colleagues isolated Atu_ph02, Atu_ph03, Atu_ph04, Atu_ph07, and Atu_ph08 with the aim of using them as biocontrol agents to treat crown gall disease. While Atu_ph08 is a temperate phage (25), the remaining four were strictly lytic and all decreased the bacterial concentration in liquid. Additionally, Atu_ph02 and Atu_ph03 diminished the number of tumors formed by tumorigenic *Agrobacterium* in a potato tumor assay (26). *Agrobacterium* phages 7-7-1 and Milano, although characterized on the molecular level, have not been tested as biocontrol agents yet (27, 28). As such, the value of *Agrobacterium* phages as a biocontrol agent remains poorly investigated. Additionally, to our knowledge, the efficacy of phage biocontrol of rhizogenic *Agrobacterium* has not yet been explored. Here, we characterized three phage species isolated from rhizogenic *Agrobacterium* biovar 1-infested tomato greenhouses and analyzed their potential as a preventive biological control agent *in vitro*.

## RESULTS AND DISCUSSION

### Isolation and taxonomic and microbiological characterization of the rhizogenic *Agrobacterium*-specific OLIVR phages demonstrates their suitability as biocontrol agents.

**FIG 1:**
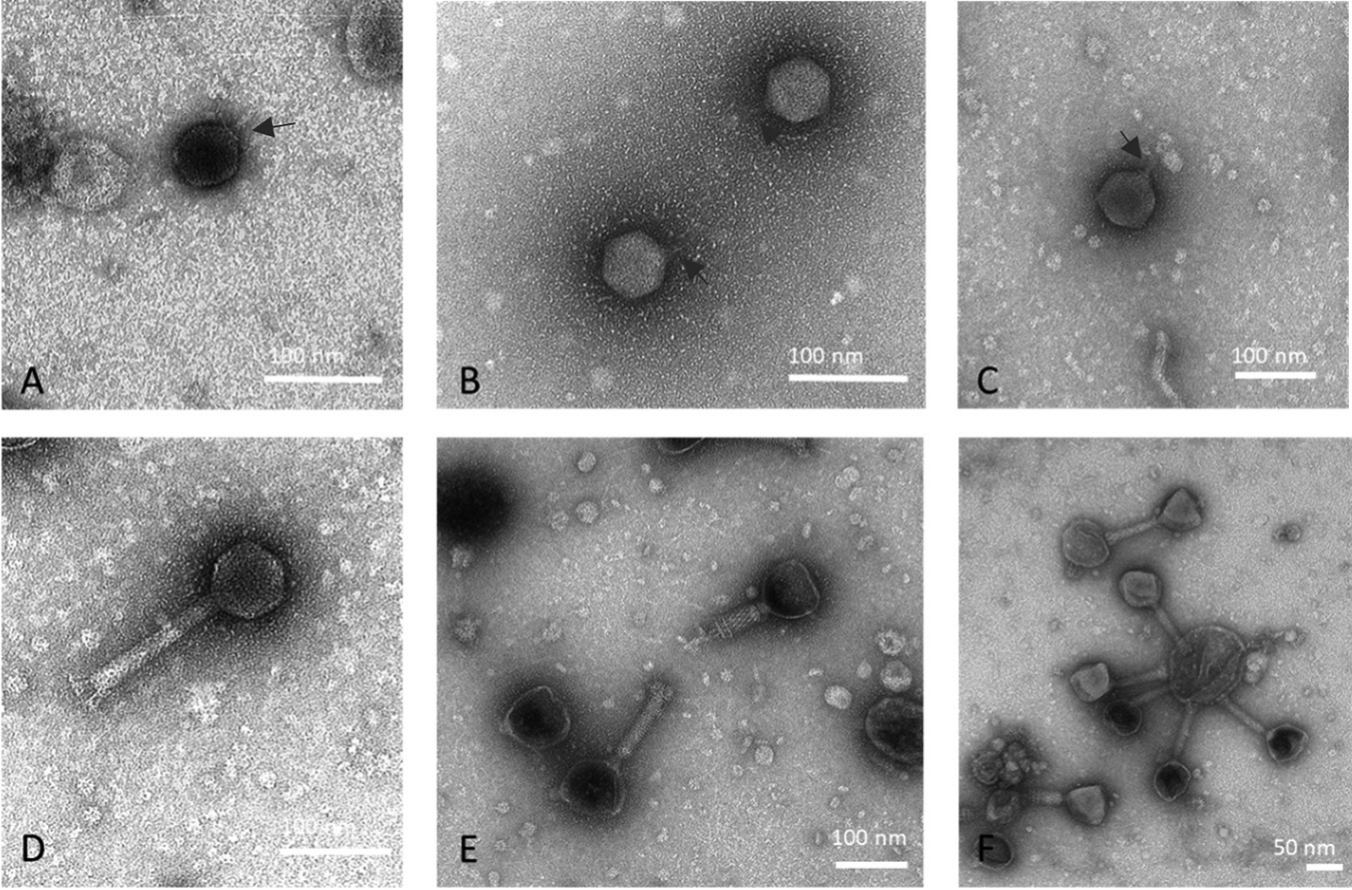
Transmission electron microscopy of six *Agrobacterium* phages. (A to C) OLIVR1 to 3 are small podoviruses, the tails of which are marked by arrows. (D) OLIVR4 is a myovirus with a rigid tail and spiky tail fibers. (E and F) OLIVR5 and 6 are myoviruses with a complex baseplate structure.

Samples were collected from tomato greenhouses in Flanders, Belgium, containing plants with abnormal root proliferation. Phages were isolated after enrichment on a random subset of strains with different phenotypes as determined by Bosmans et al. (29). Six phages were isolated and named OLIVR1 to 4, with isolation host strain ST15.13/040, and OLIVR5 and -6, with isolation host ST15.13/095, referring to Onze-Lieve-Vrouw-Waver, the site of sampling. Transmission electron microscopy (TEM) analysis shows that OLIVR1 to 3 have short noncontractile tails, whereas OLIVR4 to 6 contain long contractile tails (Fig. 1).

**FIG 2:**
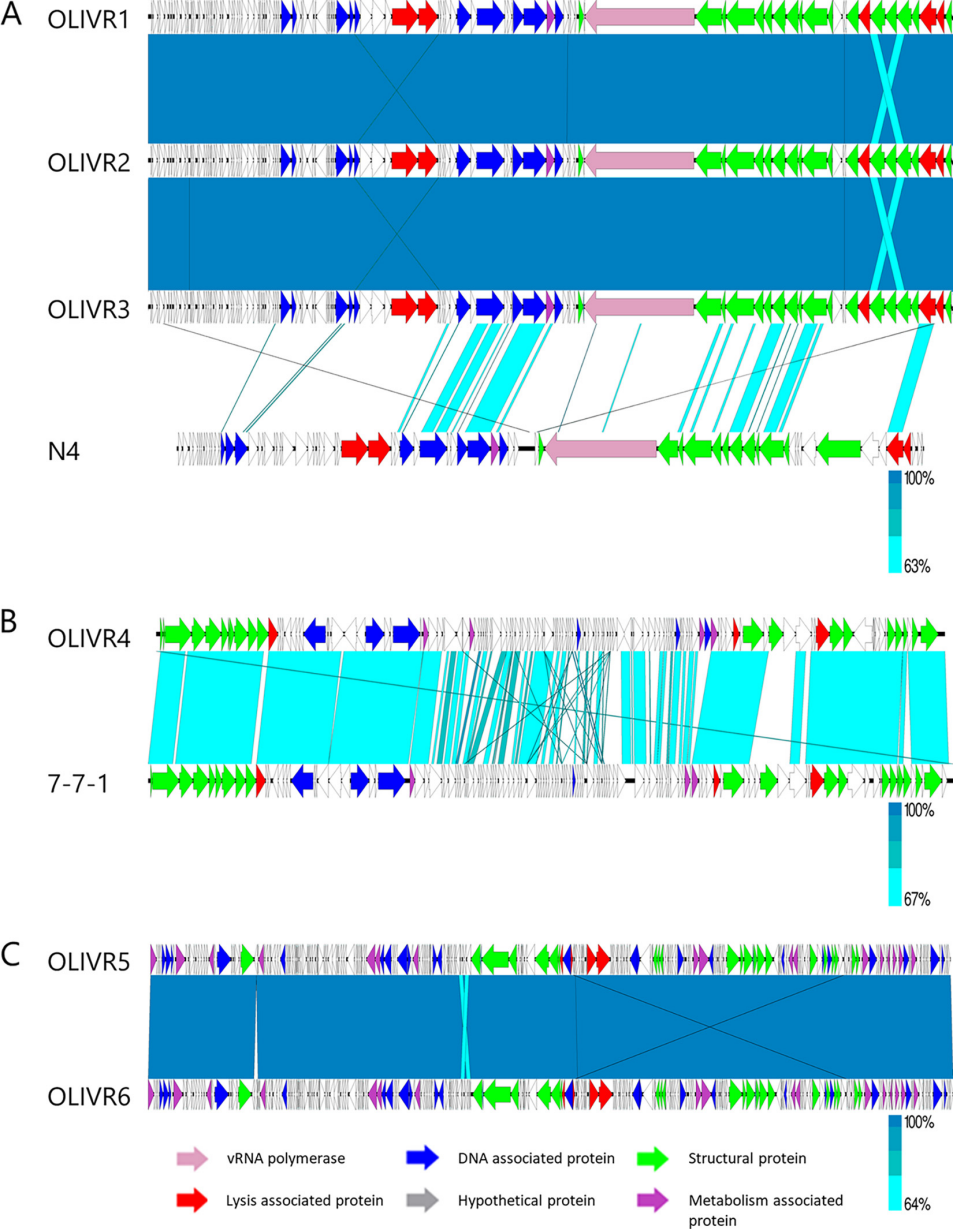
Genome maps of OLIVR1 to 6. (A) Genome maps of OLIVR1 to 3 compared to Escherichia coli phage N4. As can be seen, the genome architecture between the phages is highly conserved. (B) Genome maps of OLIVR4 compared to the closely related *Agrobacterium* phage 7-7-1. (C) The genome maps of OLIVR5 and OLIVR6 indicate that the two phages are quite similar, except for an additional region in the genome of OLIVR6 compared to OLIVR5. Genes encoding hypothetical proteins are presented as white arrows, genes encoding DNA-associated proteins as blue ones, metabolism-associated proteins in purple, lysis-associated proteins in red, and structural proteins in green.

Comparative genomics confirmed that the OLIVR phages can be classified in three different clades. More specifically, OLIVR1, OLIVR2, and OLIVR3 belong to the family *Schitoviridae*, as they have a similar genome architecture, resembling that of Escherichia coli phage N4, and encode the characteristic single-stranded DNA (ssDNA)-binding proteins (ssDB) and virion-associated RNA polymerase (vRNAP) (Fig. 2). They have a total genome size of around 75.5 kb, encoding a total of 108 proteins along with 4 tRNAs. They show the highest sequence similarity to *Delftia* phage RG-2014. However, their phylogenetic distance (<70%) (see Fig. A.1 in File S1 in the supplemental material) suggests they are members of a species from a new and distinct genus of phages (30), the *Oliverunavirus*, as established by the International Committee for the Taxonomy of Viruses (ICTV). This is based on alignments of both the vRNAP and major capsid protein (Fig. A.2 in File S1). Analysis of the protein sequence of the large terminase subunit shows that the OLIVR1 species uses short direct terminal repeat (DTR) sequences for DNA packaging (Fig. A.3 in File S1). However, since the Nextera DNA Flex library prep kit was used, these ends are not represented in the genome maps of the phages.

OLIVR4 has a genome size of 67.9 kb, encoding 131 open reading frames (ORFs) and no tRNAs. A VIRIDIC analysis shows it is most closely related to *Agrobacterium* phages Milano and 7-7-1, sharing 63.7% and 63.0% sequence identity, respectively (Fig. A.4 in File S1). Currently, these phages are considered members of the *Schmittlotzvirus* genus of an unclassified family. Analysis of the large terminase subunit shows that OLIVR4 employs a headful packaging strategy (Fig. A.3 in File S1) (31).

Finally, OLIVR5 and OLIVR6 are distantly related to orphan phages *Sinorhizobium* phage phiM9 and *Rhizobium* phage RleM_P10VF, sharing only 11% sequence identity. OLIVR5 and OLIVR6 share 99.7% sequence identity and therefore belong to the same species. Based on a VIRIDIC genome comparison (Fig. A.5 in File S1), a new (sub)family containing these phages could be proposed, mirroring the *Tevenvirinae* and *Eucampyvirinae* taxa. This newly proposed taxon would contain *Oliverocinquevirus*, as ratified by the ICTV, as a new genus (32). This is further supported by an alignment of the major capsid protein and large terminase (Fig. A.3 and Fig. A.6 in File S1). According to the terminase alignment, OLIVR5 and 6 use headful packaging (31).

No signs of a temperate life cycle were detected in any of the OLIVR phages, making them valid biocontrol candidates. This was confirmed by manual inspection and by using PhagAI which predicted them to be strictly lytic (32).

**FIG 3:**
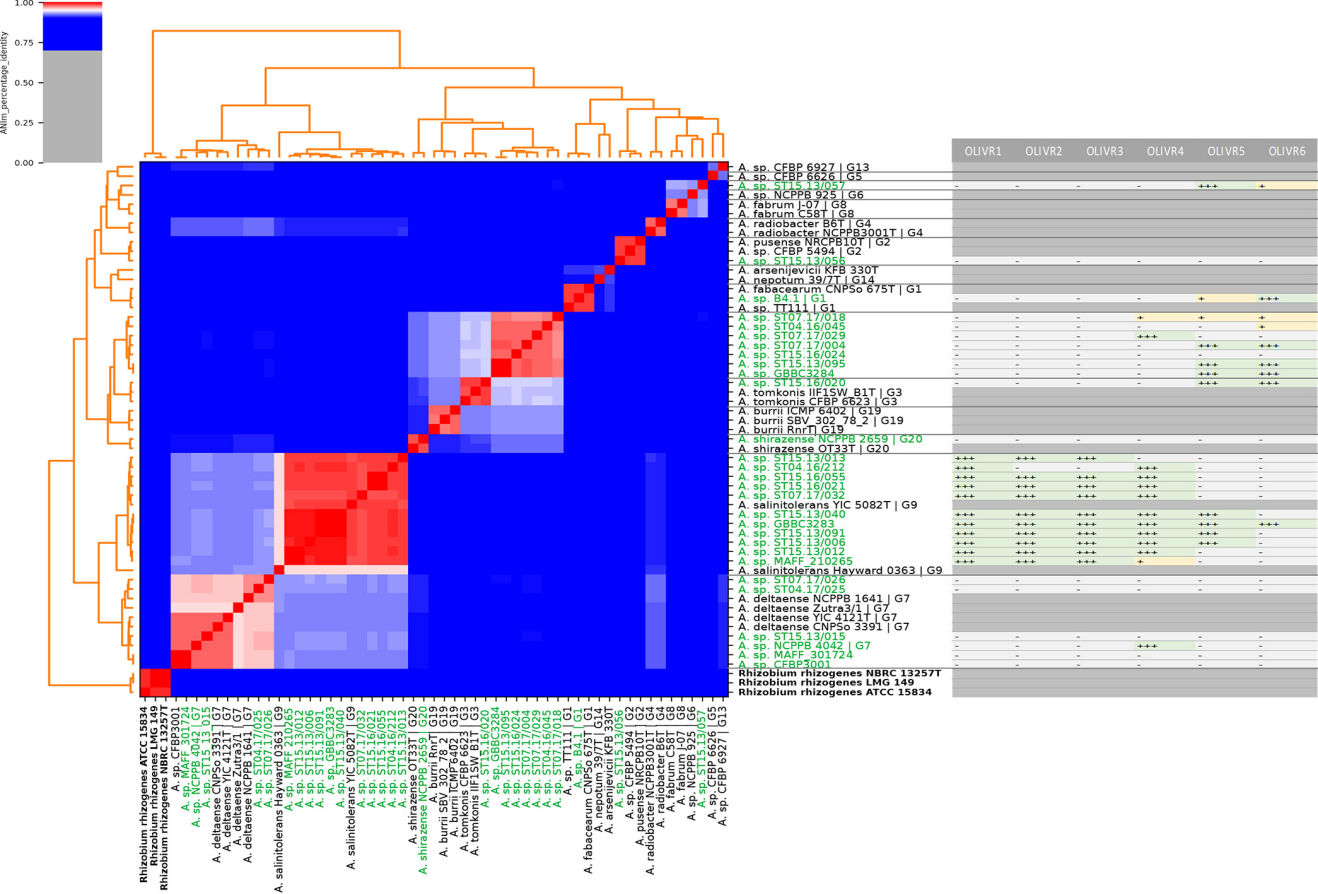
Phylogenetic analysis based on average nucleotide identity (ANI) distances between the different *Agrobacterium* strains tested in this study and their susceptibility to the OLIVR phages. Red cells in the heatmap correspond to ANIm values of 95% and higher, hence defining the same species. Blue cells show values lower than 95% and hence define different species. Color intensity fades as the comparisons approach 95% identity. The names of strains from our collection are indicated in green, while reference strains are shown in black. An outgroup of *Agrobacterium* biovar 2 strains, which are currently classified as *Rhizobium*, was used and is shown in bold. Next to each strain, its susceptibility to all OLIVR phages is summarized. A “+++” (green) shows the strains a phage could efficiently infect; a “+” shows strain could be infected by the phage, albeit with an efficiency of plating (EOP) of 0.01 or lower; a “–” (gray) shows that a phage is unable to infect the given strains. None of our strains belonged to genomospecies 4, 5, 6, 8, 13, 14, or 19. Hence these strains are not included in the host range analysis.

To assess the applicability of the phages to control rhizogenic agrobacteria, a host range analysis was performed on the entire collection of strains, which is representative for the population of agrobacteria residing in Flemish greenhouses (Table 1) (29). A comparative genomics analysis (Fig. 3) shows that all these isolates belong to *Agrobacterium* biovar 1 genomospecies (threshold, 95%; average nucleotide identity values based on MUMmer algorithm (ANIm) identity [33]). The strains belong to eight different genomospecies, including six previously described genomospecies (1, 2, 3, 7, 9, and 20). Interestingly, strain ST15.13/057 cannot be assigned to any genomospecies but is most closely related to genomospecies 6 and 8 type strains. The same is true for strains ST07.17/018, ST04.16/045, ST07.17/029, ST07.17/004, ST15.16/024, ST15.13/095, and GBBC 3284 (Fig. 3), which form a different genomospecies, which is most closely related to genomospecies 3.

**TABLE 1:** Overview of the *Agrobacterium* strain collection used

Acquisition no.	Plant host	Geographic origin	Reference or source^*a*^
MAFF210265	Cucumis melo	Japan	MAFF
NCPPB 4042	Cucumis sativus	UK	NCPPB
ST15.13/015	Solanum lycopersicum	Belgium	29
ST04.16/045	*S. lycopersicum*	Switzerland	29
ST04.16/212	*S. lycopersicum*	Belgium	29
ST04.17/025	*S. lycopersicum*	Belgium	29
ST07.17/004	*S. lycopersicum*	Belgium	29
ST07.17/018	*S. lycopersicum*	Belgium	29
ST07.17/026	*S. lycopersicum*	Belgium	29
ST07.17/029	*S. lycopersicum*	Belgium	29
ST07.17/032	*S. lycopersicum*	Belgium	29
ST15.13/006	*S. lycopersicum*	Belgium	29
ST15.13/013	*S. lycopersicum*	Belgium	29
ST15.13/056	*S. lycopersicum*	Belgium	29
ST15.13/057	*S. lycopersicum*	Belgium	29
ST15.13/091	*S. lycopersicum*	Belgium	29
ST15.16/020	*S. lycopersicum*	Belgium	29
ST15.16/021	*S. lycopersicum*	Belgium	29
ST15.16/024	*S. lycopersicum*	Belgium	29
ST15.16/055	*S. lycopersicum*	Belgium	29
CFBP3001	*C. melo*	Japan	CFBP
NCPPB 2659	*C. sativus*	UK	NCPPB
ST15.13/040	*S. lycopersicum*	Belgium	29
ST15.13/095	*S. lycopersicum*	Belgium	29
ST15.13/057	*S. lycopersicum*	Belgium	29
MAFF301724	*C. melo*	Japan	MAFF
GBBC3283	*S. lycopersicum*	Belgium	ILVO
GBBC3284	*S. lycopersicum*	Belgium	ILVO
B4.1	*S. lycopersicum*	Belgium	ILVO

a NCPPB, National Collection of Plant Pathogenic Bacteria, Harpenden, UK; MAFF, Ministry of Agriculture, Forestry, and Fisheries, Tsukuba, Ibaraki, Japan; CFBP, Collection Française des Bactéries Phytopathogènes, Institut National de la Recherche Agronomique, Beaucouzé Cedex, France; ILVO, Institute for Agricultural and Fisheries and Food Research, Merelbeke, Belgium.

This high diversity is consistent with previous results, which show that the Flemish *Agrobacterium* population responsible for hairy root disease (HRD) outbreaks is genetically very diverse (29). OLIVR1-3 had the narrowest host range, able to only infect strains from genomospecies G9. OLIVR4, on the other hand, infected strains from genomospecies G3, G7, and G9, whereas OLIVR5 infected strains from G1, G3, G9, and the two yet unassigned genomospecies (including amongst others strains ST15.13/057 and ST07.17/018). As such, our current phage collection can infect strains from a total of six genomospecies, accounting for 75% of the strain collection.

OLIVR1, OLIVR4, and OLIVR5 were selected for further microbiological characterization, since they were the isolates with the broadest host range for the different phage species. First, we evaluated the phages’ adsorption rate to their isolation host. OLIVR1 (Fig. A.7A in File S1) adsorbed the fastest, as 99.9% of the added phages adsorbed within the first minute after inoculation. Based on an adsorption efficiency model (34), the adsorption constant for OLIVR1 is 5.23 × 10^−8 ^mL/min. OLIVR4 (Fig. A.7B in File S1) and OLIVR5 (Fig. A.7C in File S1) were characterized by a much slower adsorption rate, as both phages required approximately 50 min to adsorb. Their adsorption kinetics are therefore best described using a sequential model (34). As such, OLIVR4 and OLIVR5 have an adsorption rate constant of 3.02 × 10^−10 ^mL/min and 4.11 × 10^−10 ^mL/min, respectively.

**FIG 4:**
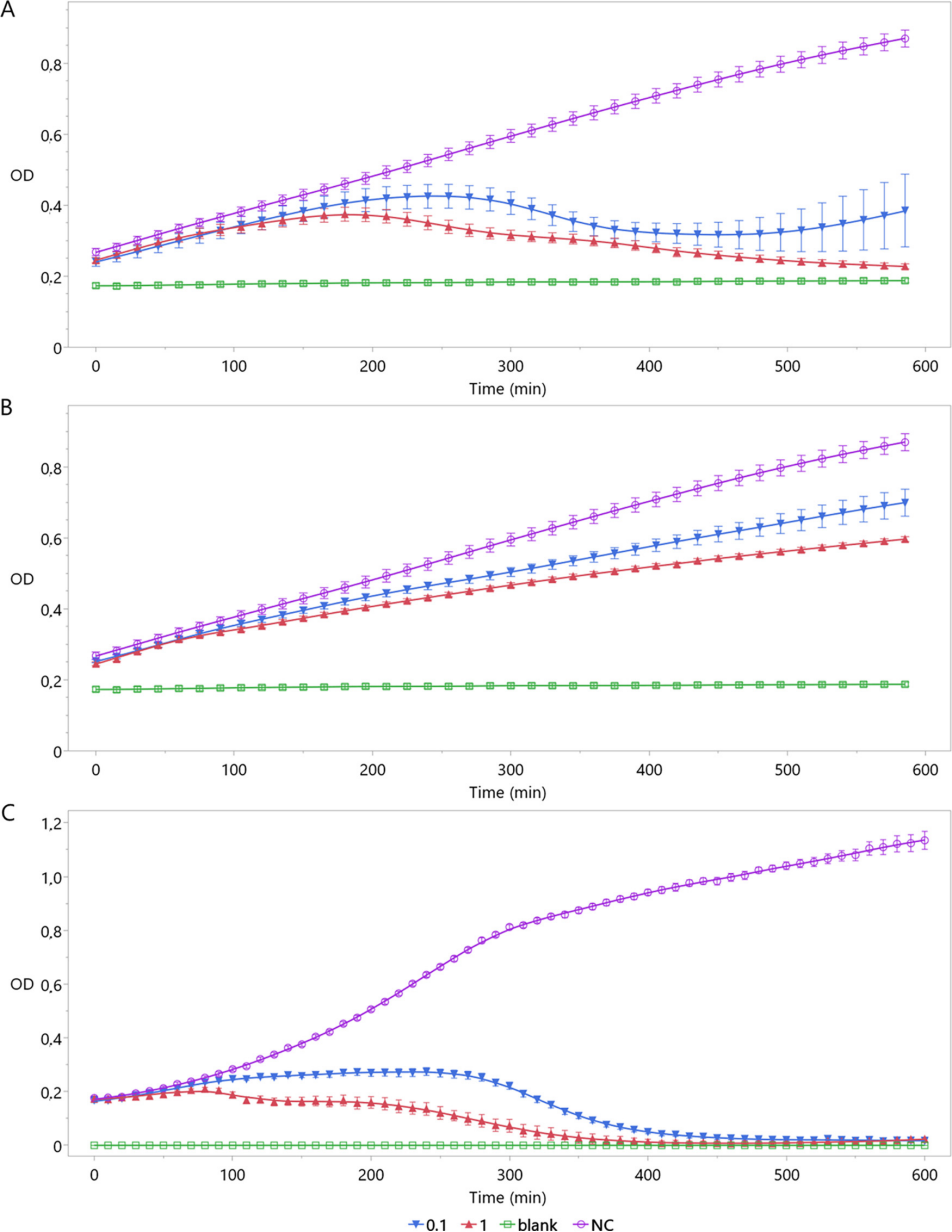
(A to C) Infection curves of OLIVR1 (A), OLIVR4 (B), and OLIVR5 (C). For the negative control (NC) without phage (purple), the bacterial concentration continuously increases over time. When OLIVR1 (A) is used to infect ST15.13/040, the bacterial population declines. OLIVR4 (B), on the other hand, can only reduce the growth speed of this strain. Just like OLIVR1, OLIVR5 (C) succeeds at decreasing the bacterial concentration of its host, ST15.13/095, but in contrast to OLIVR1, no new resistant population emerged.

Next, the *in vitro* lysis activity of the phages was analyzed (Fig. 4). At a multiplicity of infection (MOI) of 1 and 0.1, OLIVR1-infected cultures started to decline after 3 to 4 h, respectively. In the case of OLIVR5, the bacterial population declined 80 min after infection at an MOI of 1 and after 4 h at an MOI of 0.1. Interestingly, no new resistant bacterial population emerged within 10 h after infection, in contrast to OLIVR1. Contrary to OLIVR1 and OLIVR5, OLIVR4 did not show a pronounced lysis activity *in vitro* but, rather, slowed down the growth of the bacterium. This might be explained by the difference in adsorption rate, as faster-adsorbing phages tend to display a higher impact on bacterial concentrations (35).

Subsequently, two transposon knockout libraries were created of host strains ST15.13/040 (host of OLIVR1 and OLIVR4) and ST15.13/095 (host of OLIVR5) to determine the phage receptors. These libraries consisted of 10^5^ transformants for both strains, thus representing the whole bacterial genome. Table 2 summarizes the mutants that gained phage resistance with an efficiency of plating (EOP) lower than 0.01 compared to the wild-type host.

**TABLE 2:** Summary of the gene products that are impaired due to the integration of a Tn*5* transposon, causing phage resistance

Phage	Strain	Protein	Accession no.
OLIVR1	Tn5OL1R2	Pentapeptide repeat-containing protein	WP_142911795.1
	Tn5OL1R4	Flagellar type III secretion system protein FliR	WP_003503350.1
	Tn5OL1R5	Recombinase family protein	WP_142911796.1
	Tn5OL1R6	NYN domain-containing protein	WP_003496951.1
	Tn5OL1R10	NYN domain-containing protein	WP_003496951.1
OLIVR4	Tn5OL4R1	Flagellar biosynthesis repressor FlbT	WP_080826090.1
	Tn5OL4R2	Flagellar biosynthesis protein FlhA	AHK00443.1
	Tn5OL4R3	Flagellar biosynthesis repressor FlbT	WP_080826090.1
	Tn5OL4R4	Flagellar type III secretion system protein FliR	WP_003503350.1
	Tn5OL4R6	Flagellar biosynthesis protein FlhA	AHK00443.1
	Tn5OL4R8	Flagellar biosynthesis repressor FlbT	WP_080826090.1
OLIVR5	Tn5OL5R2	alpha-d-glucose phosphate-specific phosphoglucomutase (ExoC)	WP_142842470.1
	Tn5OL5R3	Pyrimidine utilization protein B (RutB)	WP_065114118.1
	Tn5OL5R4	Hypothetical protein	WP_142841699.1
	Tn5OL5R5	Amino acid ABC transporter permease (HisM)	WP_065117267.1
	Tn5OL5R5	Sensor histidine kinase protein (BaeS)	ANM14467.1
	Tn5OL5R6	ATP-binding cassette, subfamily F member 3 (Uup)	SOD52793.1
	Tn5OL5R6	Fumarylacetoacetate hydrolase family protein	WP_142842196.1
	Tn5OL5R7	Sensor histidine kinase protein (BaeS)	ANM14467.1

OLIVR1-resistant clones contained the transposon in genes encoding either the flagellar type III secretion system protein (FliR), an NYN domain-containing protein, a pentapeptide repeat-containing protein, or a recombinase family protein. FliR is a part of the inner membrane complex, involved in the export of flagellar proteins (36). The NYN domains are typical folds of nucleases that play roles in central cellular processes, i.e., DNA replication and repair, noncoding RNA (ncRNA) maturation, transcription regulation, and mRNA degradation (37). The C-terminal end of the pentapeptide repeat-containing protein, though, has some sequence similarity to YjbH, a protein involved in the regulation of virulence gene expression and bacterial surface structures and conferring resistance to oxidative stress (38).

The importance of FliR was also demonstrated for OLIVR4. In addition, FlhA and FlbT are key for OLIVR4 infection. While FlhA is responsible for the formation of an inner membrane import complex forming a nonameric ring structure, FlbT is a transcription regulator (class III) that regulates the production of flagellin polypeptides (39, 40). Based on phylogeny, the relationship between phage infectivity and the presence of a functioning flagellum is further demonstrated. The closest relative of OLIVR4, *Agrobacterium* phage 7-7-1, is known to be a flagellotrophic phage and requires actively rotating flagella to infect its host (41). As such, we hypothesize that OLIVR4 requires flagella for infection as well.

The transposon knockout strategy was more ambiguous for OLIVR5. Resistant clones Tn5OL5R2, Tn5OL5R4, and Tn5OL5R7 contain one transposon insertion, while the other mutants carry transposons at different loci across the genome (Table 2).

Finally, the stability of the OLIVR phages under cultivation-relevant conditions was determined in tomato nutrient solution (plant growth medium for tomato [PGMT]) and in the presence of hydrogen peroxide. None of the phages were inactivated in PGMT over a period of 10 days (Fig. A.8 in File S1). However, the tolerance to hydrogen peroxide varied between the phages (Fig. A.9 in File S1). The least stable phage was OLIVR1, which was inactivated at all applied concentrations. The higher the hydrogen peroxide concentration, the more pronounced the degradation of phage particles. After 1 day, no OLIVR1 phage particles were recovered from 100 and 300 ppm hydrogen peroxide. In 30 and 60 ppm, phages were recovered, but the titer was significantly lower than for the control (*P* < 0.001). The same was true after 5 days, with OLIVR1 showing a log 1.5 reduction at 30 ppm and a log 2.6 reduction at 60 ppm. After 10 days, no active phages were recovered under the conditions with hydrogen peroxide. OLIVR5 showed higher stability than OLIVR1 and proved stable for all hydrogen peroxide concentrations after 1 day. However, significant differences were observed after 5 days. Only the condition with 30 ppm H_2_O_2_ did not show a difference from the negative control. Active phages could still be recovered from 60 and 100 ppm, but not from 300 ppm. The concentration of phage in 30 ppm H_2_O_2_ also became significantly lower after 10 days. OLIVR4 showed the highest stability to hydrogen peroxide. Only after 10 days were the differences compared to the negative control significant. The concentration of phage was slightly lower for all conditions with H_2_O_2_, with the lowest concentration in 300 ppm.

### The OLIVR phages show different disinfection efficiencies in PGMT.

The potential to disinfect using phages was tested by adding 10^8^ PFU/mL to inoculated tomato nutrient solution. Two different bacterial loads were used: 10^2^ CFU/mL, which corresponds to the observed concentrations in an average infested greenhouse, and 10^4^ CFU/mL, corresponding to more heavily infested greenhouses toward the end of the season (42). This assay could be translated in practice as a prevention strategy, which is necessary because *Agrobacterium* colonizes the roots of its hosts, after which it injects its transfer DNA (T-DNA), causing an irreversible hormonal imbalance and an induction of pathogenicity within the circulating agrobacterium population. Hence, the pathogenic load in the nutrient solution must be reduced to a minimum to avoid this. To make sure that as many cells as possible were lysed, a high MOI was chosen. This approach, called passive treatment, has already been put forward as the most promising for phage therapy in human applications (43, 44). The bacterial concentration was measured at the start of the experiment and at 1 and 7 days after phage addition. The phage titer was also monitored during this period and was shown to remain constant during the entire experiment (Fig. A.10 in File S1).

**FIG 5:**
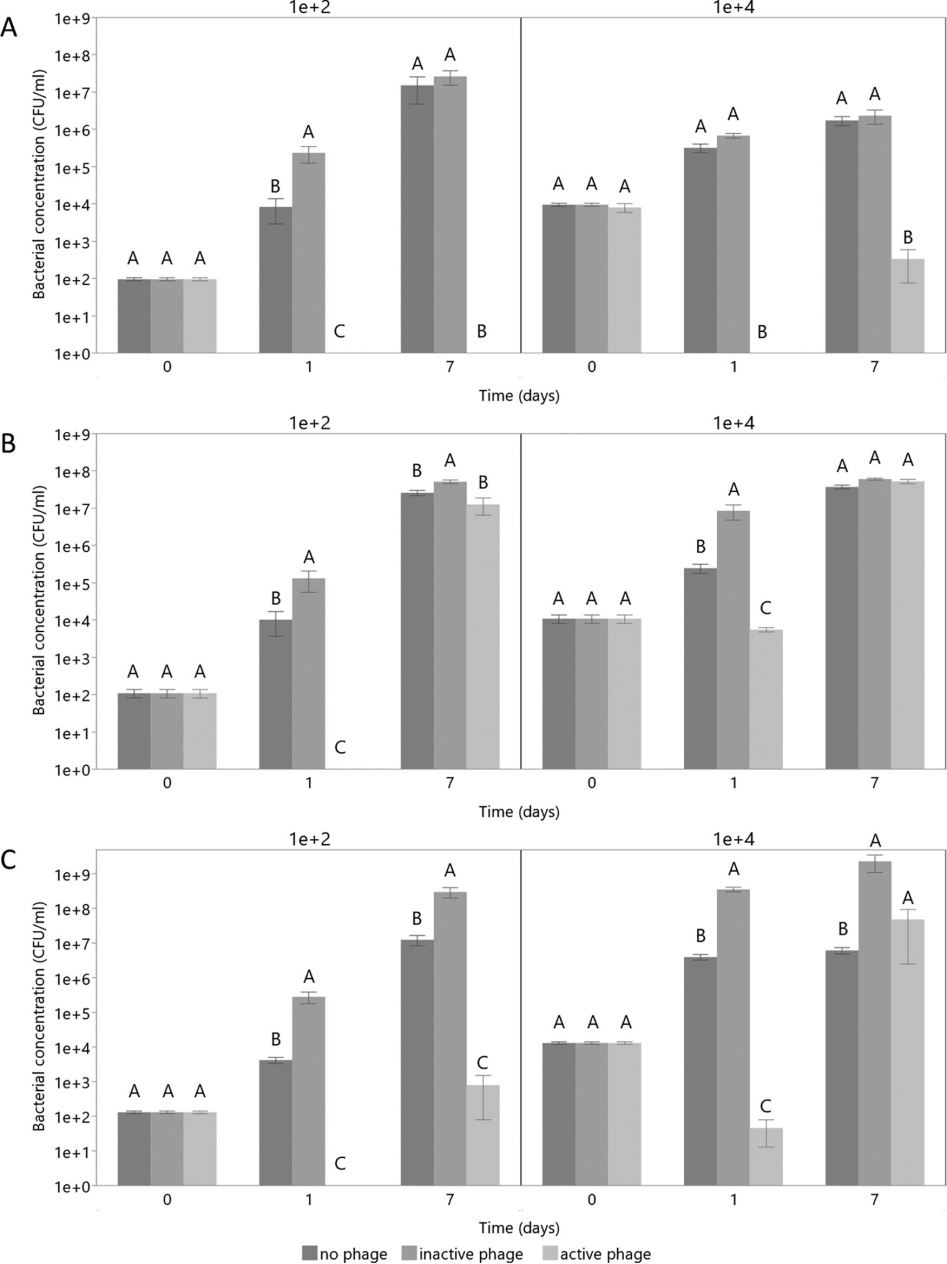
Disinfection of *Agrobacterium*-contaminated PGMT using the OLIVRs. (A to C) Progress of the disinfection assay with (A) OLIVR1, (B) OLIVR4, and (C) OLIVR5. The *y* axis shows the average bacterial concentration (CFU/mL) of five biological repeats, while the error bars represent the standard error. All phages were tested using a bacterial load of 10^2^ (left part of graphs) and 10^4^ CFU/mL (right part of graphs). All phages succeeded at infecting their host in the nutrient solution but showed different efficiencies, with OLIVR1 being the only phage able to decrease the bacteria below the limit of detection. The connecting letters show which values of the same days were significantly different (Steel-Dwass test, α = 0.05). For each start concentration, five biological repeats were used.

All phages can infect their host in PGMT. However, OLIVR1 was the most efficient at clearing the nutrient solution from bacterial cells (Fig. 5). This phage killed the bacteria below the detection limit (LOD) for the low-bacterial-load sample, while bacterial concentration increases in the controls. The population size was kept below the LOD after 7 days, while the controls without phage and with inactivated phage reached titers of above 10^7^ CFU/mL. Upon disinfecting PGMT with a higher bacterial load, similar results were obtained. A final concentration of 3 × 10^2^ CFU/mL was reached for the phage-treated group, while for the controls, the final concentrations reached approximately 10^6^ CFU/mL. As observed during the killing curve experiments, OLIVR4 was the least efficient. It reduced the bacterial load below the LOD when using 10^2^ CFU/mL. For the higher bacterial load, however, no significant decrease was observed. After 7 days, all conditions reached titers of 10^7^ CFU/mL or higher. OLIVR5 displayed a disinfection efficiency lower than that of OLIVR1 but higher than that of OLIVR4. This phage killed the bacteria below the LOD for the low bacterial load. Upon disinfecting PGMT with 10^4^ CFU/mL, the bacterial population after 1 day was reduced by log 2.6 but remained above the detection limit. For the 10^2^ CFU/mL sample, the population size increased to 8 × 10^2^ CFU/mL after 7 days. For the higher initial load, a final concentration of 5 × 10^7^ CFU/mL was reached for the phage-disinfected group.

Based on this assay, OLIVR1 and OLIVR5 appear to be the most promising phages, as they reduce the bacterial load. OLIVR4, on the other hand, shows more of a growth speed-decreasing effect. The observed bacterial reductions are similar to previous trials with Pseudomonas aeruginosa, Ralstonia solanacearum, and *Vibrio* species (45–47). OLIVR4, on the other hand, failed to decrease the bacterial load significantly but had a growth speed-reducing effect. Possibly, this might again be explained by the low adsorption efficiency of OLIVR4, as phages with a low adsorption speed do not always have a great reduction effect on the bacterial concentration (48). Although this phage appears less promising, it could maybe be used in combination with the other OLIVR phages. Another possibility is to combine it with other treatments such as hydrogen peroxide or other biocontrol organisms.

### Identification of mutations that lead to phage resistance.

Clones surviving the disinfection treatment were evaluated for their sensitivity toward the phages. None of the clones picked after 7 days of treatment with OLIVR1 (for the high-bacterial-load sample), showed resistance to OLIVR1. Although this seems counterintuitive, it is possible that the bacteria have a gene expression-based mechanism that was expressed during the disinfection assay but not after reisolation. Indeed, some sensitive bacteria can live alongside their phage as a result of phenotypic resistance (49).

All colonies isolated from nutrient solution treated with OLIVR4 or OLIVR5 had developed actual resistance. No cross-resistance was observed against the other phage species. Three of the OLIVR4-resistant mutants, OL4R1, OL4R2 and OL4R3, and three of the OLIVR5 resistant mutants, OL5R1, OL5R2, and OL5R3, were selected for further characterization.

First, their genomes were sequenced to determine mutations (Table 3). For OL4R1 and OL4R3, single nucleotide polymorphisms (SNPs) were identified in an intergenic region, whereas for OL4R2, a mutation occurred within *rcsC*, *acrB*, and a gene encoding a ferredoxin NADP^+^ reductase. In addition, a deletion causing a frameshift was found in a region encoding a hypothetical gene. RcsC is a sensor histidine kinase. In E. coli, this protein controls the transcription of numerous genes, including those involved in colanic acid capsule synthesis, biofilm formation, and cell division (50). AcrB is a component of an export pump, which in E. coli is part of the AcrA-AcrB-AcrZ-TolC drug efflux system (51). Ferredoxin NADP^+^ reductase catalyzes the reduction of NADP^+^ to NADPH using ferredoxin and can serve in various metabolic pathways (52).

**TABLE 3:** Summary of mutations in the OLIVR4- and OLIVR5-resistant mutants

Isolate	Gene affected	Type of mutation	Effect of mutation
OL4R1	Intergenic region	Transition (T:C)	Unknown
OL4R2	*rcsC*	Transition (A:G)	Phe140Leu
	*acrB*	Transition (A:G)	Thr301Ala
	Ferredoxin NADP^+^ reductase	Transition (A:G)	Cys281Arg
	Hypothetical gene	Deletion (TG:T)	Gly122fs
OL4R3	Intergenic region	Transition (A:G)	Unknown
OL5R1	UDP-glucose 4-epimerase (*galE*)	Transition (C:T)	Arg222His
OL5R2	*ropA1* and *ropA2*	Large deletion	Recombination
OL5R3	UDP-glucose 4-epimerase (*galE*)	Transition (C:T)	Arg222His

For the OLIVR5-resistant clones, the same SNP was found in OL5R1 and OL5R3 in *galE*, encoding a UDP-glucose-4-epimerase, which catalyzes the synthesis of UDP-galactose from UDP-glucose (53). Several *Agrobacterium* and other *Rhizobiaceae* species use galactose as an important building block for their lipopolysaccharides (LPS) (54), and the importance of *galE* in LPS and exopolymeric substance (EPS) synthesis has been studied thoroughly in different bacterial genera (55–57). For OL5R2, no SNPs could be identified. Instead, OL5R2 had lost a fragment of 1,203 bp ranging from position 678,687 to 679,890 on the ST15.13/095 genome. Interestingly, this fragment encompasses a portion of two gene homologues present in all *Rhizobiales*, including *Agrobacterium*: *ropA1* and *ropA2.* Both encode a putative outer membrane porin (58). The deletion resulted in the loss of (i) five nucleotides of *ropA1*, including its start codon, (ii) the entire fragment between *ropA1* and *ropA2*, and (iii) 589 nucleotides of *ropA2*, including its stop codon. As such, both genes had recombined into a new one, without causing a frameshift.

Next, adsorption tests were performed to determine whether the phages could still bind to the bacterial surface of these resistant mutants. The transposon mutants obtained during the receptor analysis (Tn5OL4R8, Tn5OL5R2, Tn5OL5R6, and Tn5OL5R7) were included for comparison (Fig. A.11 and A.12 in Text S1). An F-test (α = 0.05) showed that the phage titer did not decrease over time, while the opposite was true for the wild-type bacterium. Hence, there was no adsorption to the resistant mutants from the disinfection assay, nor to the mutants of the receptor analysis. As such, these data show that both the spontaneous mutations from the *in vitro* disinfection assay and the transposon insertions caused resistance by receptor modification.

At first sight, it might seem counterintuitive that the OLIVR4-resistant mutants from the disinfection assay do not have mutations in genes associated with flagellar function. However, phage 7-7-1 requires LPS alongside flagella to adsorb (41). Hence, the same is potentially true for OLIVR4. For OLIVR5, on the other hand, the mutations are more straightforward in their interpretation. Indeed, its closest relative, Sinorhizobium meliloti phage ΦM9 is known to recognize LPS moieties as a receptor. As such, it is likely that the mutation in *galE* has changed the LPS of the resistant strains, blocking adsorption. Similarly, S. meliloti phage ΦM9 and at least 10 other phages recognize RopA1 as a receptor. As such, the same is likely true for OLIVR5, explaining why the recombination between *ropA1* and *ropA2* blocks adsorption (58).

### Investigation of the fitness cost associated with phage resistance.

**FIG 6:**
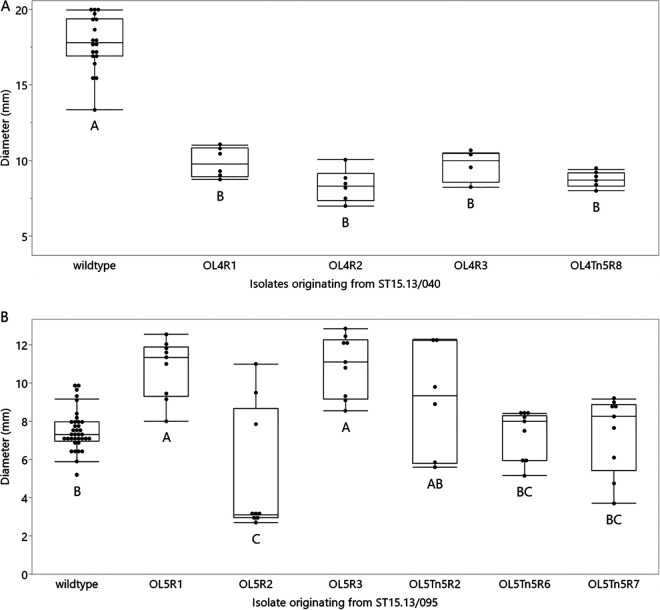
Motility assay for phage-resistant mutants. (A) The motility of OLIVR4-resistant mutants was compared to the wild-type phage-sensitive strain. As can be seen in the connecting letters report, the wild type-sensitive isolate had a significantly higher motility than the resistant ones. (B) Motility of OLIVR5 resistant mutants obtained during the disinfection assay and during receptor analysis compared to the wild-type phage-sensitive strain. Statistically significant differences were found, but different effects were observed, and the effect on motility was different for each isolate. Two independent trials were performed, each with three biological repeats.

Since the receptor analysis of OLIVR4 pointed toward the flagella as a candidate receptor and because of its homology to the flagellotrophic *Agrobacterium* phage 7-7-1, it was hypothesized that the OLIVR4-resistant mutants had a reduced swimming motility. As the receptor of OLIVR5 was more elusive, it was hypothesized that the motility of resistant mutants would remain unaffected. Indeed, all OLIVR4-resistant mutants show a decreased motility (Tukey-Kramer test; α = 0.05; *P* < 0.0001) (Fig. 6). For OLIVR5, differences in motility are also observed (Fig. 6). However, in contrast to OLIVR4-resistant isolates, there was no clear decrease in motility. Indeed, phage resistance seemed neither to decrease nor increase motility, but the effect was different for different isolates.

Finally, to assess if resistance was correlated with a loss of virulence, a bean virulence assay was set up. All resistant isolates originating from strain ST15.13/040 as the ancestral strain had the ability to cause hairy root development, except for Tn5OL4R8 (Fig. A.13 in Text S1). The resistant isolates of ancestral strain ST15.13/095 also retained the ability to cause disease, without exception. Hence, contrary to many other studies, which showed that phage-resistant bacteria had reduced virulence, no virulence cost was observed in this assay (59–63). However, since the OLIVR4-resistant isolates have impaired motility and some of the OLIVR5-resistant isolates from the water disinfection assay have mutations in genes involved in LPS production, the virulence of these isolates might be affected if the bacterium needs to swim to and colonize the plant independently (64).

### Prospects for a sustainable phage-based treatment against hairy root disease.

Hairy root disease is a rapidly spreading disease worldwide in commercial greenhouses that rely on hydroponics. This waterborne disease affects tomatoes, eggplant, cucumber, and bell pepper and is mostly caused by a diversity of *Agrobacterium* biovar 1 genomospecies. We have isolated six phages belonging to three different clades that can infect a total of six genomospecies out of the eight genomospecies present in our collection. Due to the waterborne nature of the disease, we tested the effectivity of the phages to specifically kill *Agrobacterium* in PGMT. OLIVR5 and OLIVR1 were the most efficient phages at decreasing the bacterial concentration of artificially infected PGMT. These results are in line with previous trials in which water contaminated by P. aeruginosa, R. solanacearum, or *Vibrio* species was disinfected (45–47). However, it must be noted that OLIVR1, although being very effective at decreasing the bacterial load without phage resistance emerging, only infected a single genomospecies of our collection. For OLIVR5, resistance did emerge, causing a mutation in *galE*, a gene involved in LPS synthesis, and *ropA1* and *ropA2*, both encoding outer membrane structures. OLIVR4, on the other hand, failed at significantly reducing the bacterial load after a day of incubation with *Agrobacterium*, underscoring the importance of testing phages separately for a strategy. However, all bacteria isolated after incubation with the phages had become resistant to the phages, and hence the gene pool of bacteria had changed. This resistance was accompanied by a decrease in motility which potentially constrains *Agrobacterium*’s ability to compete with other bacteria for nutrients or its ability to translocate to and colonize their host’s roots. As such, this phage may still be useful in real greenhouses. Additionally, contemporary disease control today has shifted toward an integrated pest management approach. In this approach, multiple suited techniques are combined to prevent economic losses due to pests. It can be generally stated that the use of phages such as OLIVR1, OLIVR4, and OLIVR5 could readily be introduced in an integrated pest management approach, as the specific nature of phages and their overall stability are traits that would support this. For instance, it might be interesting to combine the OLIVR phages with the *Paenibacillus* strains of Bosmans and colleagues.

## MATERIALS AND METHODS

### Phage isolation, amplification, and purification.

A set of representative *Agrobacterium* strains (29) (Table 1) was grown in lysogeny broth (LB) at 25°C. Using these strains as baits, phages OLIVR1 to 6 were isolated from 0.5 g wet rockwool samples containing roots, collected in 2019 from three infected commercial tomato greenhouses in Flanders, Belgium. For phage amplification, host strains were grown to the early exponential growth phase (optical density at 600 nm [OD_600_] of 0.3), as previously described (65). Next, phages were added at a multiplicity of infection (MOI; this is the phage-bacterium ratio) of 0.01. *Agrobacterium* strain ST15.13/040 was used to amplify phages OLIVR1 to 4, while ST15.13/095 was used for OLIVR5 and -6. The culture was incubated overnight at 25°C with shaking at 200 rpm. The culture was centrifuged (4°C, 4,000 × *g*, 1 h) and filtered using Nalgene Rapid-Flow filter units of 500 to 1,000 mL with a 0.45-μm polyethersulfone (PES) membrane. When needed for DNA extraction, phages were precipitated with polyethylene glycol (PEG 8000) by incubating the phage suspension with 15% ice-cold PEG overnight before centrifugation (4°C, 4,000 × *g*, 1 h).

### Phage microbiological characterization.

Transmission electron microscopy (TEM) was performed as described previously (66). Phage lysis activity was tracked over time by infecting an exponentially growing bacterial culture with phages (MOI of 0.1 to 1) and measuring the OD_600_ over time. The adsorption of the phages on their bacterial host was measured by following the concentration of free phage over time and comparing it to the initial concentration, as previously described (67). For this, a bacterial culture with an OD_600_ of 0.3 (1.25 × 10^8^ CFU/mL) was infected at an MOI of 0.01, after which samples were taken at different time points based on which phage was used. Chloroform was added to these time samples, after which they were briefly centrifuged. Next, the titer was determined by a double agar overlay assay. In the final step, the adsorption coefficient was calculated according to Storms and Sauvageau (34).

The host range of the phages on the collection bacterial strains (Table 1) was evaluated using a drop test. Here, 3 μL of phage serial dilutions (10^6^ to 10^5^ to 10^4^ PFU/mL) was spotted on a bacterial lawn. When individual plaques were observed, the strain was considered susceptible to the specific phage. Finally, the stability of the phages was assessed by incubating a phage dilution of 10^8^ PFU/mL under different conditions: in a concentration range of hydrogen peroxide (30, 60, 100, and 300 ppm) and in plant growth medium for tomato (PGMT) collected from the greenhouses at KU Leuven (Belgium) (PGMT: rain water supplied with 0.642 mM Ca(NO_3_)_2_ · 4H_2_O, 0.325 mM KNO_3_, 0.45% C_10_H_12_FeN_2_O_8_, 0.195 mM K_2_SO_4_, 0.195 mM MgSO_4_ · 7H_2_O, 0.195 mM KH_2_PO_4_, 0.00001 mM (NH_4_)_6_Mo_7_O_24_ · 4H_2_O, 0.0002 mM ZnSO_4_ · 7H_2_O, 0.00007 mM CuSO_4_ · 5H_2_O, 0.00455 mM MnSO_4_ · H_2_O, and 0.00325 mM H_3_BO_3_). Phages were counted by plating after specific time intervals depending on the assay performed. This was done with five biological repeats.

### Receptor analysis.

An *Agrobacterium* transposon knockout library was generated using the EZ-Tn5 <KAN-2>Tnp Transposome kit (Epicentre, Lucigen; Middleton, WI, USA). Briefly, *Agrobacterium* strains ST15.13/040 and ST15.13/095, grown to an OD_600_ of 0.5 and washed with ice-cold glycerol 10%, were electroporated (12.5 kV/cm; 25 μF; 200 Ω) (46), plated on selective LB medium (50 μg/mL kanamycin), and incubated for 2 days at 25°C. Colonies were pooled and diluted to a final concentration of 10^8^ CFU/mL. To select for phage-resistant clones (against OLIVR1, OLIVR4, or OLIVR5, individually), the knockout mutant library was infected with an MOI of 50 and incubated for 2 days at 25°C on LB agar with kanamycin (50 μg/mL). Growing colonies were picked, inoculated in liquid selective medium, and reevaluated for their resistance by spotting 5 × 10^7^ PFU on top of a bacterial lawn containing the presumably phage-resistant clone. As such, clones that showed resistance to the phages were selected for further analysis. Cross-resistance of these knockout mutants to the other phages was again tested by spotting 3 μL of a dilution series (10^6^ to 10^5^ to 10^4^ PFU/mL). DNA was extracted using the GeneJET genomic DNA purification kit (Thermo Fisher Scientific, Waltham, MA, USA). The location of the transposon inside the genome of the phage-resistant clones was determined by means of a thermal asymmetric interlaced PCR (TAIL-PCR) as previously described (68). The flanking regions of the transposon were Sanger sequenced and analyzed using a tBLASTx search (69).

### DNA sequencing and phylogenetic analysis.

Bacterial genomic DNA was extracted using the DNeasy UltraClean microbial kit (Qiagen) and phage DNA by phenol-chloroform extraction (70). Bacterial and phage genomes were sequenced using an in-house MiniSeq Illumina platform. The Nextera Flex DNA library kit was used for the library prep of the DNA. The average length (700 bp) of the DNA fragments was evaluated using an Agilent Bioanalyzer 2100 and a high-sensitivity DNA kit (Agilent Technologies). The concentration was determined on a Qubit instrument (Thermo Fisher Scientific). Demultiplexed and trimmed paired reads were directly generated by the Illumina MiniSeq. The Bacterial and Viral Bioinformatics Resource Center (BV-BRC) platform was used to assemble these paired reads using SPAdes as the assembly strategy (71). Next, the assembled bacterial genomes were annotated using BV-BRC (domain *Bacteria*; taxonomy ID 359; default settings). Eventually, pyANI (72) (v0.2.11) was used to compute the average nucleotide identity with ANIm as the method, to determine the relatedness of the strain collection. Additionally, genomes from reference strains were included to assign strains to a certain genomospecies (Table 4). Biovar 2 strains, currently classified as *Rhizobium*, were included as an outgroup. The assembled phage genomes were also annotated using BV-BRC (domain *Virus*; taxonomy ID 357; phage settings), after which similar phages were identified using ViPTree (v1.9) and visualized using Easyfig (73, 74). To classify the phages, genomic distances between phage genomes were determined by means of a VIRIDIC analysis (75) and visualized using Seaborn (76). The genomes were checked for the presence of terminators using ARNold (77) and promoters using MEME (78) and PHIRE (79). Single nucleotide polymorphisms were checked with Snippy (80) on the Galaxy platform (v3.2) (81). Phylogenetic analysis of the phages was performed by aligning (MUSCLE) the major capsid proteins, virion-associated RNA polymerase, portal vertex protein, and/or large terminase and calculating a neighbor-joining tree based on this alignment in MEGA X with a bootstrap of 1,000 (82).

**TABLE 4:** Overview of the reference strains used for the phylogenetic analysis of the *Agrobacterium* strains^*a*^

Strain	Genomospecies	Accession no.
*Agrobacterium* sp. TT111	G1	GCA_900012575.1
Agrobacterium fabacearum CNPSo 675	G1	GCA_009649785.1
Agrobacterium pusense NRCPB10	G2	GCF_002008275.1
*Agrobacterium* sp. strain CFBP 5494	G2	GCA_900013495.1
Agrobacterium tomkonis CFBP 6623	G3	GCA_900013535.1
Agrobacterium tomkonis IIF1SW_B1	G3	JABXYF000000000
Agrobacterium radiobacter B6	G4	GCA_900045375.1
*A. radiobacter* NCPPB3001	G4	GCF_001541305.1
*Agrobacterium* sp. strain CFBP 6626	G5	GCA_900012595.1
*Agrobacterium* sp. strain NCPPB 925	G6	GCA_900012625.1
Agrobacterium deltaense CNPSo 3391	G7	GCA_003931535.1
*A. deltaense* YIC 4121	G7	GCF_002008205.1
*A. deltaense* Zutra3/1	G7	GCA_900013515.1
*A. deltaense* NCPPB 1641	G7	GCA_900012585.1
Agrobacterium fabrum C58	G8	GCA_000092025.1
*A. fabrum* J-07	G8	GCA_900013525.1
Agrobacterium salinitolerans YIC 5082	G9	GCA_002008225.1
*A. salinitolerans* Hayward 0363	G9	GCA_900012565.1
*Agrobacterium* sp. strain CFBP 6927	G13	GCA_900012615.1
Agrobacterium nepotum 39/7	G14	JWJH00000000.1
Agrobacterium burrii RnrT	G19	GCA_017313405.1
*A. burrii* SBV_302_78_2	G19	GCA_008501935.1
*A. burrii* ICMP 6402	G19	GCA_009498615.1
Agrobacterium shirazense OT33	G20	GCF_017313365.1
Rhizobium rhizogenes ATCC 15834	NA	GCA_006802085.1
Rhizobium rhizogenes LMG 149	NA	GCA_007002995.1
Rhizobium rhizogenes NBRC 13257	NA	GCA_000696095.1
Agrobacterium arsenijevicii KFB 330	NA	GCA_000949895.1

a NA, not applicable, since they are biovar strains.

### Data availability.

The bacteriophage genome sequences were deposited in NCBI under the accession numbers MT234338, MT234339, MT234340, MT234341, and MT234342. The bacterial genomes were submitted to NCBI and are available under BioProjects PRJNA893450, PRJNA914635 and PRJNA914038.

### Testing phage efficacy in a selective nutrient solution disinfection assay.

To estimate how efficiently the phages disinfect the nutrient solution in a greenhouse, *Agrobacterium* and individual phages were incubated together in PGMT. An overnight culture of the bacteria with an OD_600_ of 1 to 1.2 (approximately 10^9^ CFU/mL) was centrifuged (4,500 rpm; 10 min; 25°C), and the cell pellet was resuspended in sterile (autoclaved) PGMT. This procedure was repeated twice to remove any remaining nutrients from the LB. Next, a 10-fold dilution series was made from the resulting bacterial suspension using PGMT, and the bacterial concentration was determined by plating. This was done with five independent biological repeats. The dilutions of 10^2^ and 10^4^ CFU/mL received 10^8^ PFU/mL of OLIVR1, OLIVR4 (both using strain ST15.13/040), or OLIVR5 (added to ST15.13/095). Controls without active phage received an equal volume of phage buffer (10 mM Tris · HCl, pH 7.5, 10 mM MgSO_4_, 150 mM NaCl) or inactivated (autoclaved) phage lysate. The phage titer and bacterial titer were determined by spotting. Controls with only phage and sterile PGMT were also included, and all conditions were incubated at 25°C. The bacterial and phage concentration was tracked on days 1 and 7 after incubation. At the end of the experiment, colonies were picked from the samples that received phages and those that did not. These isolates’ phage susceptibility was determined as described above. These clones were also used further to determine their characteristics and genome. Next, Snippy (80) was used to check for mutations that introduced phage resistance, while Bowtie 2 (83) on the Galaxy platform (81) was used to align the reads to the genome of the ancestral strain to check for larger deletions. To estimate how efficiently the phages disinfect the nutrient solution in a greenhouse, *Agrobacterium* and individual phages were incubated together in PGMT. An overnight culture of the bacteria with an OD_600_ of 1 to 1.2 (approximately 10^9^ CFU/mL) was centrifuged (4,500 rpm; 10 min; 25°C), and the cell pellet was resuspended in sterile (autoclaved) PGMT. This procedure was repeated twice to remove any residual nutrients from the LB. Next, a 10-fold dilution series was made from the resulting bacterial suspension using PGMT, and the bacterial concentration was determined by plating. This was performed in 5-fold. The dilutions of 10^2^ and 10^4^ CFU/mL received 10^8^ PFU/mL of OLIVR1, OLIVR4 (both using strain ST15.13/040), or OLIVR5 (added to ST15.13/095). Controls without active phage received an equal volume of phage buffer (10 mM Tris · HCl, pH 7.5, 10 mM MgSO_4_, 150 mM NaCl) or inactivated (autoclaved) phage lysate. The phage titer and bacterial titer were determined by spotting. Controls with only phage and sterile PGMT were also included, and all conditions were incubated at 25°C. The bacterial and phage concentration was tracked by plating on days 1 and 7 after incubation. At the end of the experiment, colonies were picked from the samples that received phages and those that did not. Phage susceptibility was determined as described above. These clones were also further characterized, and their genomes were sequenced. Next, Snippy (80) was used to check for small mutations that had enabled phage resistance, while Bowtie 2 (83) on the Galaxy platform (81) was used to align the reads to the genome of the ancestral strain to check for larger deletions. This allowed generation of ugenedb files that were inspected using UGENE 38.1.

### Motility assay.

A motility assay was performed to assess if resistance development of the bacteria in the nutrient solution disinfection bioassay impaired bacterial motility. For each condition, three biological repeats were prepared together with a control (wild-type strain) in two independent trials. First, single colonies were grown overnight. Next, motility was tested on 0.3% LB agar plates by dipping a sterile toothpick into the overnight culture and gently piercing the agar. Wild-type strains were dipped on the plate along with resistant strains with sufficient interspacing. After 48 h of incubation at 25°C, the size of the colony was measured using a sliding gauge ruler (Mitutoyo, Beveren, Belgium) with an accuracy of 0.5 mm.

### Virulence assays.

The virulence of selected wild-type *Agrobacterium* strains and their resistant mutants was assessed using a bean virulence assay according to Estrada-Navarrete and colleagues with minor changes (84). In short, nondisinfected seeds of the bean cultivar Prelude (Sanac, Roeselaere, Belgium) were germinated in commercial potting soil for sowing and propagation (DCM, Grobbendonk, Belgium). After 2 weeks, plants were transplanted to 1.5-L pots (three plants per pot) containing potting soil mixed with perlite (DCM, Grobbendonk, Belgium). Bacterial cultures were initiated from 15% glycerol stocks and grown on LB agar at 25°C. Colonies from a 2-day-old subculture were suspended in 10 mM phosphate buffer (PB, pH 7.0) to obtain a concentration of a minimum of 10^9^ CFU/mL (OD_600_, 1 to 1.2). The pin-prick procedure was used to inoculate the plants, applying two 5-μL drops on either side of the stem at the height of and perpendicular to the cotyledons. The droplets were punctured several times with an entomological needle to introduce the inoculum into the vascular tissue. PB served as a negative control. Inoculations were performed in triplicate (three pots each containing three plants) per tested isolate. Plants were kept at 100% humidity for 21 days, after which the roots emerging at cotyledon height were cut and weighed per pot.

### Statistical analysis.

All graphs were made and all statistical tests were performed using JMP Pro 16 (SAS Institute, Inc., Cary, NC). Before testing for significant differences, normality was assessed using a Shapiro-Wilk test (α = 0.05), and homoscedasticity was assessed using an O’Brien test (α = 0.05). When the data were normally distributed, a Tukey-Kramer test (α = 0.05) was used to compare the means between different groups. When the data were not normally distributed, a Steel-Dwass test (α = 0.05) was used.
