# Supplementary material for: Back to the Roots: Agrobacterium-Specific Phages Show Potential to Disinfect Nutrient Solution from Hydroponic Greenhouses
Source: Appl Environ Microbiol. 2023 Apr 3;89(4):e00215-23. doi: 10.1128/aem.00215-23 (PMC10132094; doi:10.1128/aem.00215-23)
Supplement: Supplemental file 1 — Supplemental material. Download aem.00215-23-s0001.pdf, PDF file, 1.0 MB [file aem.00215-23-s0001.pdf]

Appendixes

VIRIDIC analyses of OLIVR1-6

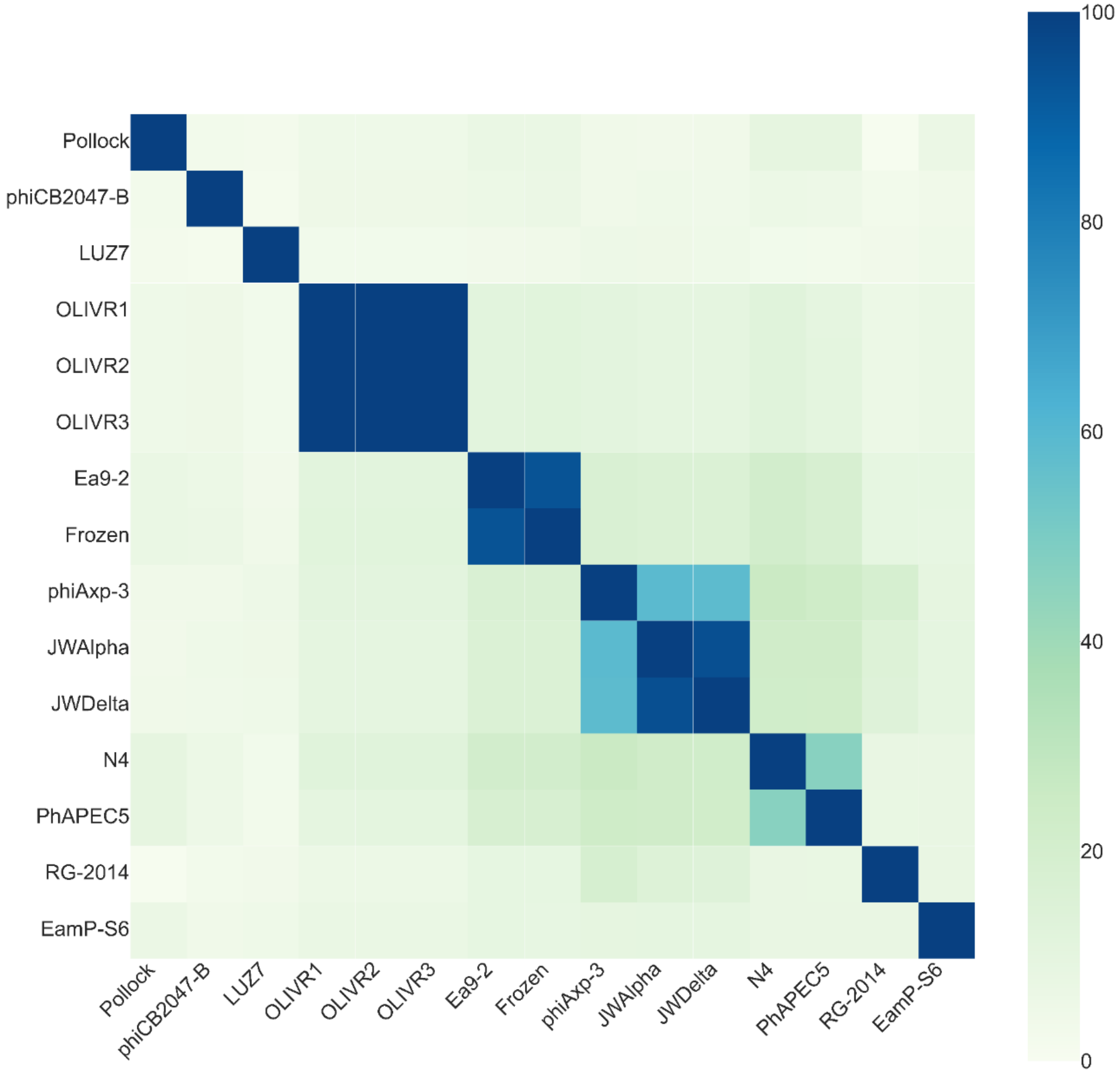

*Figure A.1: **VIRIDIC heatmap of OLIVR1-3 and its most related phages of the Schitovirdae family**. This heatmap shows the percentage identity of OLIVR1-3 with its most closely related relatives based on the protein sequence. OLIVR1-3 are most closely related to the Schitoviridae family, to which they are compared here, in which they form a new and distinct group.*

Major capsid and vRNA polymerase tree of OLIVR1-3

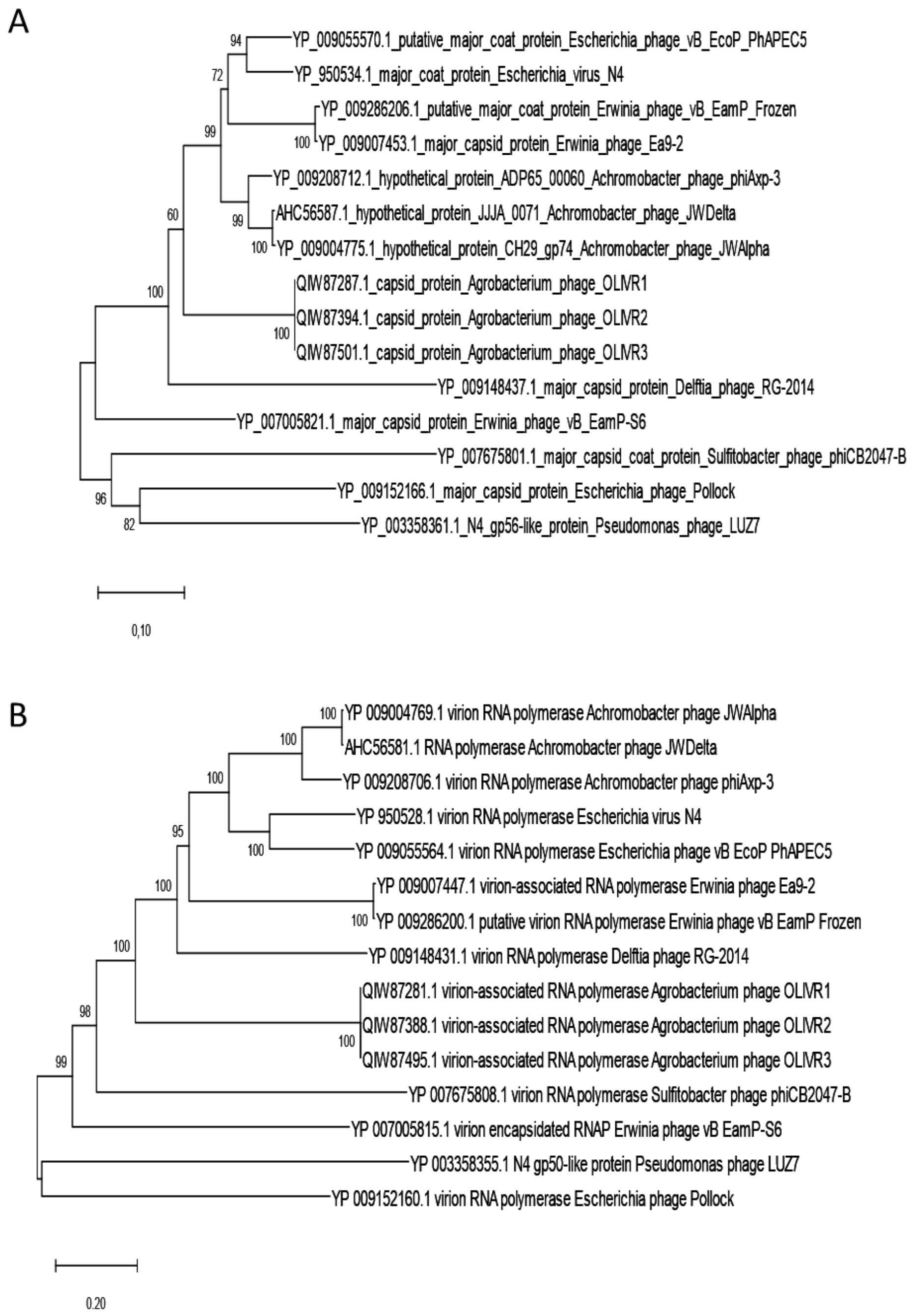

Figure A.2: Neighbour-joining trees based on the amino acid sequence of the major capsid protein (A) and vRNAP (B) confirm OLIVR1-3 are unique members in the Schitovirdae family. Bootstrap values are given above the nodes of the trees. The bar at the bottom represents the number of sustitutions per site.

Terminase tree of all OLIVRs

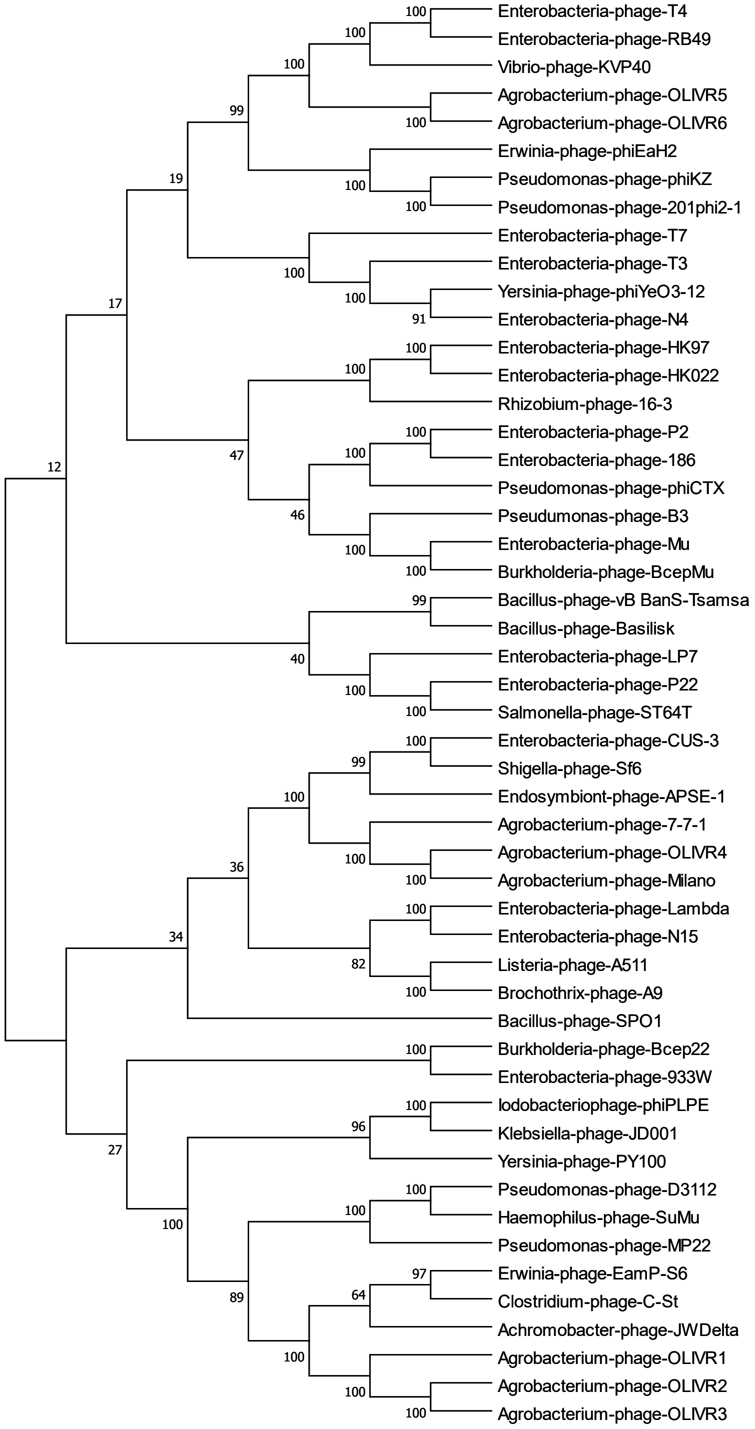

Figure A.3: A neighbour-joining tree of the large terminase subunit was generated as described by Merill and colleagues [31] *Comparison of this three with theirs allowed to identify the packaging strategies of the OLIVRs. OLIVR1-3 use small DTRs, while OLIVR4-6 use headful packaging.*

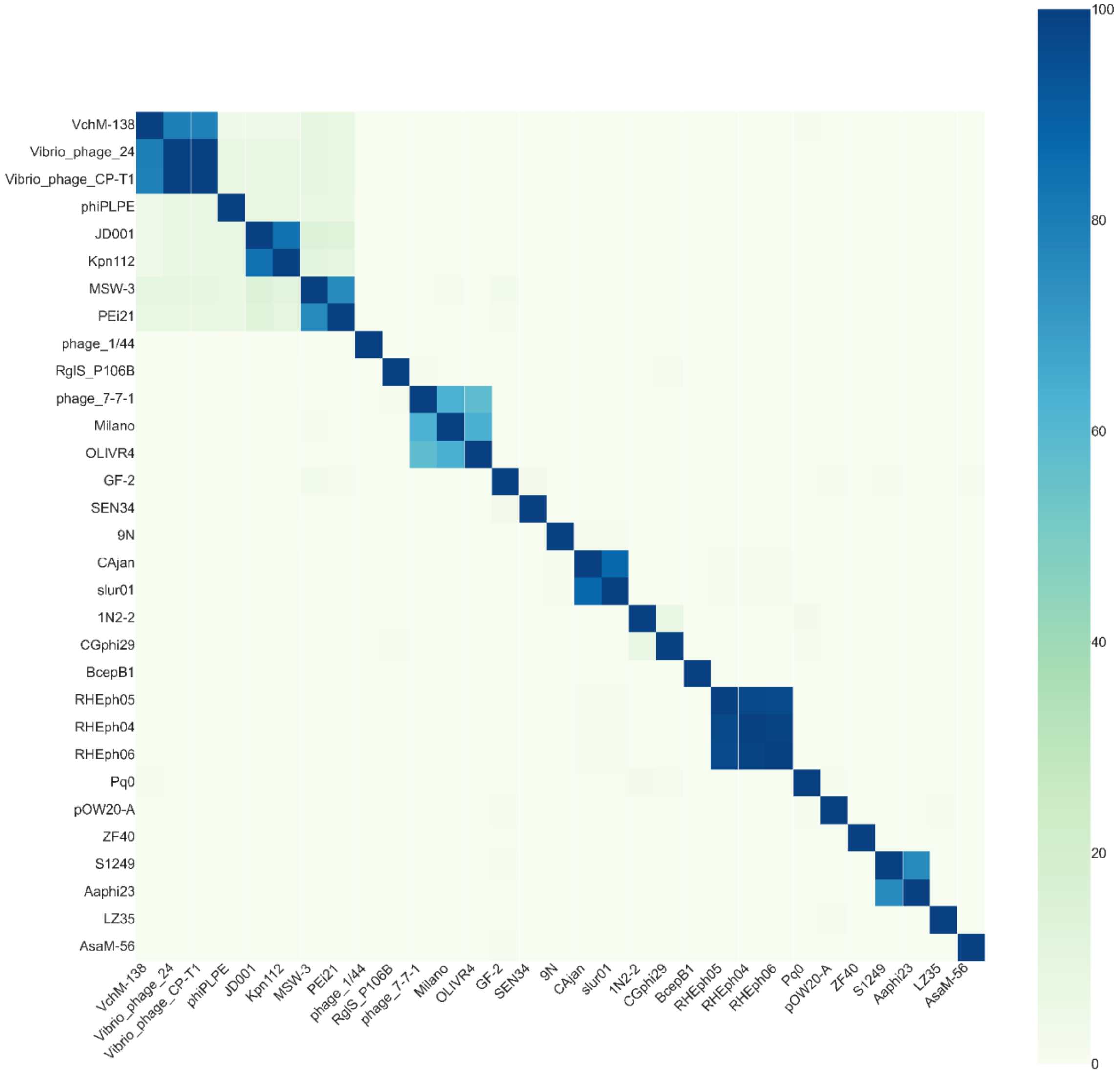

*Figure A.4: **VIRIDIC heatmap of OLIVR4 and its most related phages.** This heatmapmyoviruses that are most related to OLIVR4. OLIVR4 is most closely related to Agrobacterium phage 7-7-1 and Agrobacterium phage Milano which belong to the Schmitlotzvirus genus.*

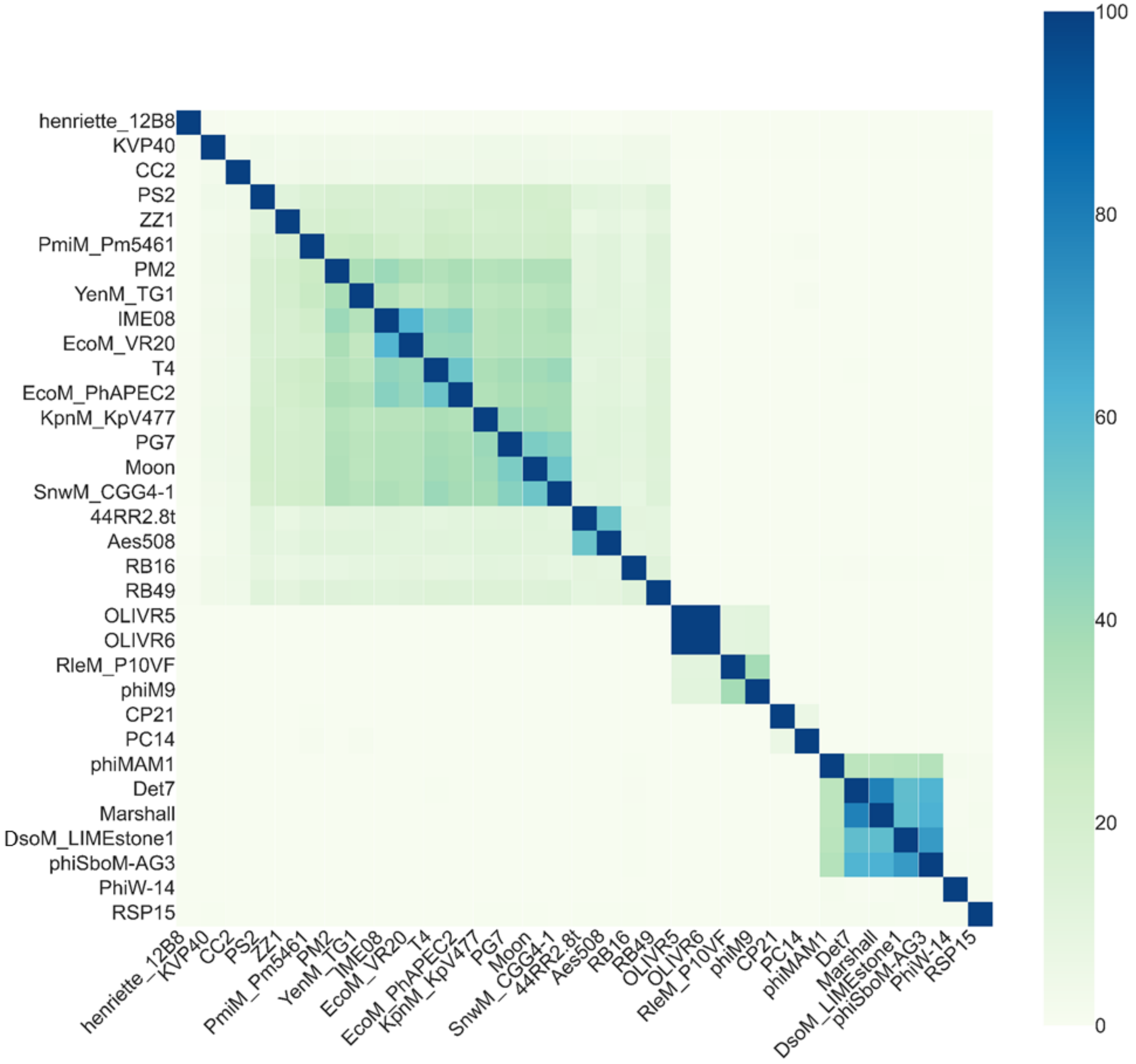

Figure A.5: : ***VIRIDIC heatmap of OLIVR5-6 and its most related members of the Myoviridae.*** *The legend shows the percentage of sequence identity. OLIVR5 and OLIVR6 show relatedness with Rhizobium phage RlcM_P10VF and Sinorhizobium phage phiM9. This cluster is quite isolated from the two neighboring clusters known as the Tevenvirinae (PS2 till RB49) and the Eucampyvirinae (CP21 and PC14).*

Major capsid and portal vertex protein of OLIVR5-6

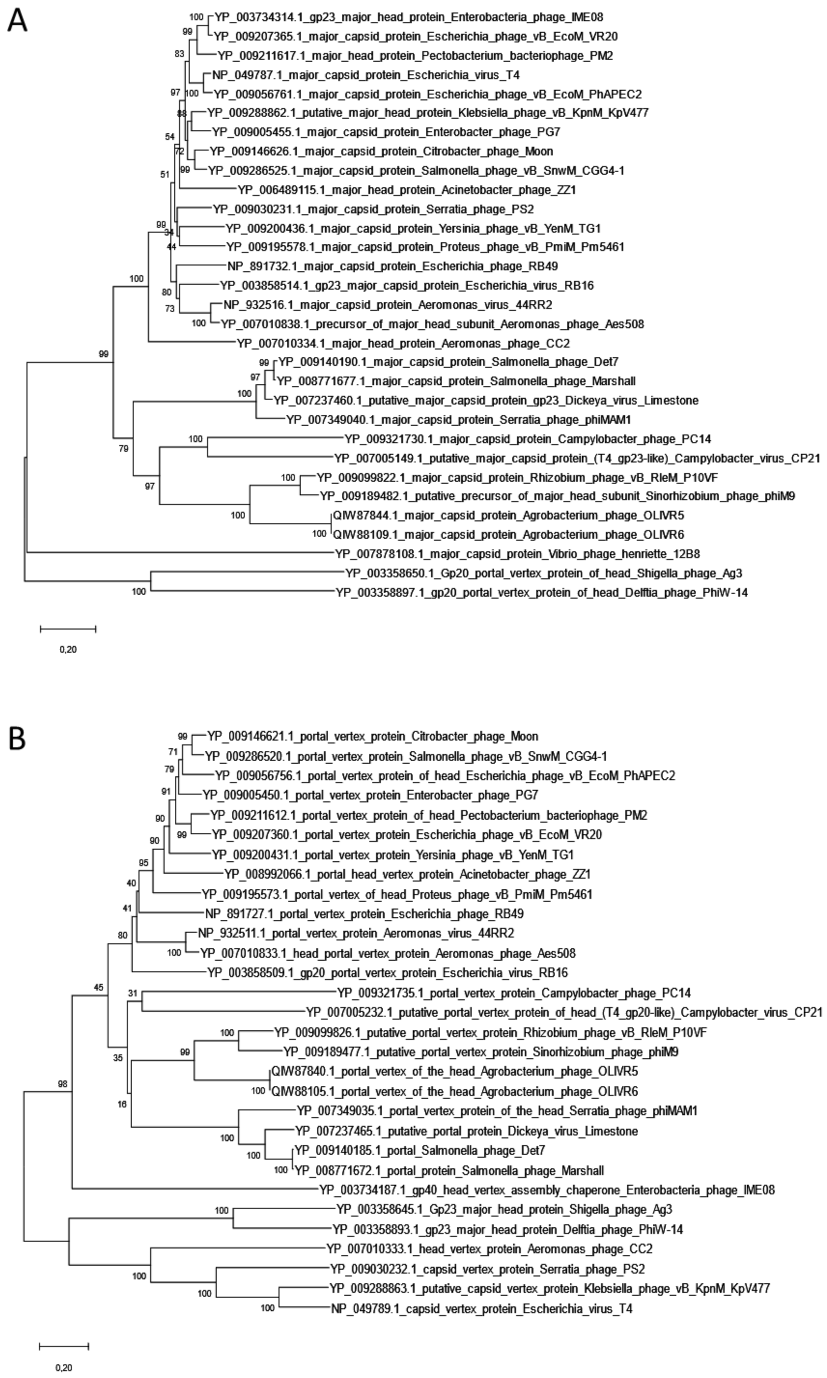

Figure A.6: (A) Neighbour-joining trees based on the amino acid sequence of the major capsid protein and (B) the portal vertex protein, respectively. These trees show the relatedness to Rhizobium phage RlcM_P10VF and SinoRhizobium phage phiM9 as well. Bootstrap values are indicated above the nodes (1.000 bootstraps). The bar at the bottom represents the number of sustitutions per site.

Adsorption curves on host strains.

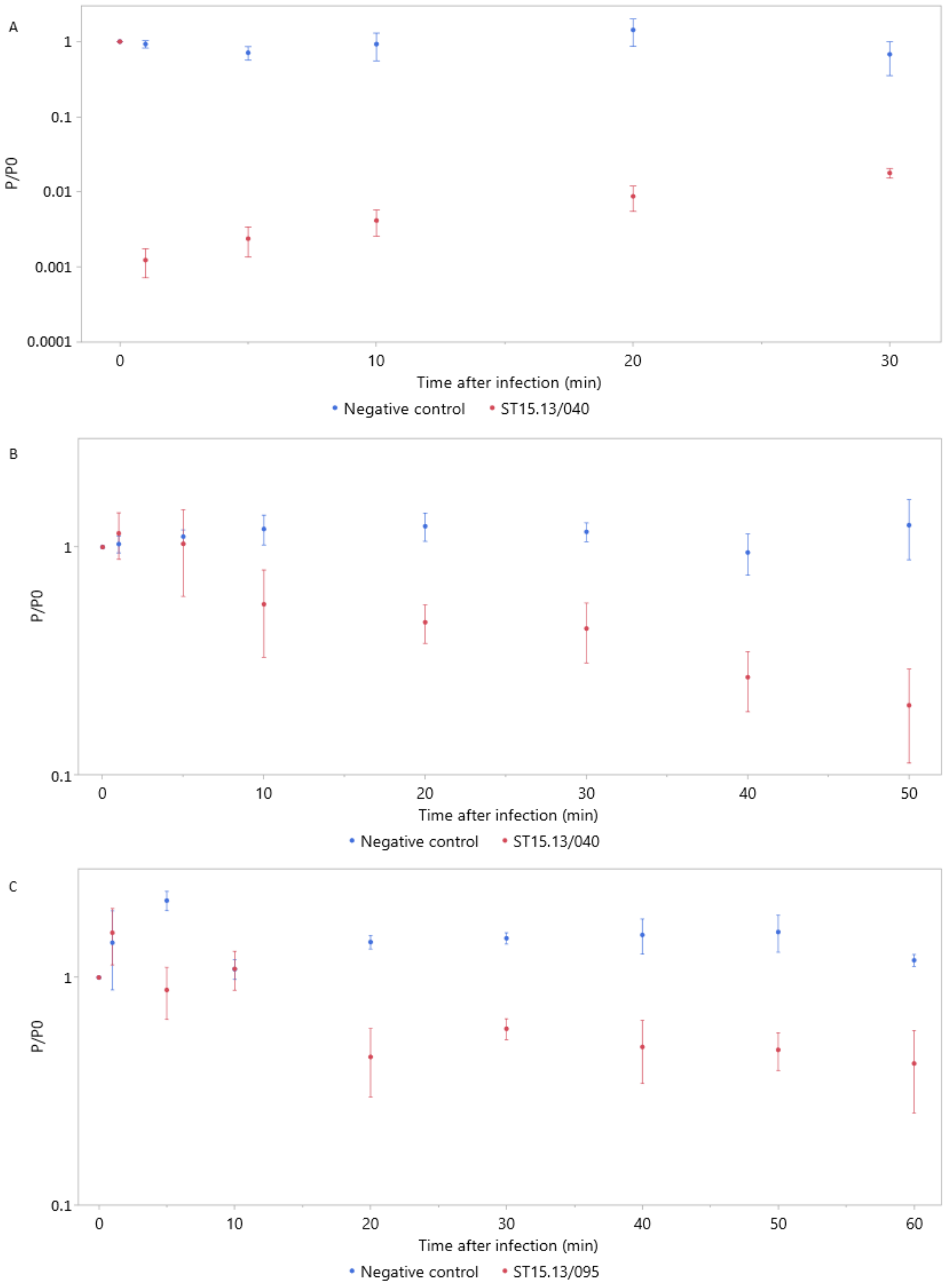

*Figure A.7: **Adsorption curves of the OLIVR phages**. (A) OLIVR1 was the fastest adsorbing phage that followed an adsorption efficiency model with an adsorption rate constant of 5.23 · 10^{-8}ml/min. Hence within 1 min, all phages were adsorbed. (B) OLIVR4 kinetics were best described with a sequential adsorption model with an adsorption rate constant of 3.015 · 10^{-10} ml/min and (C) OLIVR5 were best described with a sequential adsorption model with an adsorption rate constant of 4.110 · 10^{-10} ml/min.*

Stability of the OLIVR-phages in greenhouse relevant conditions

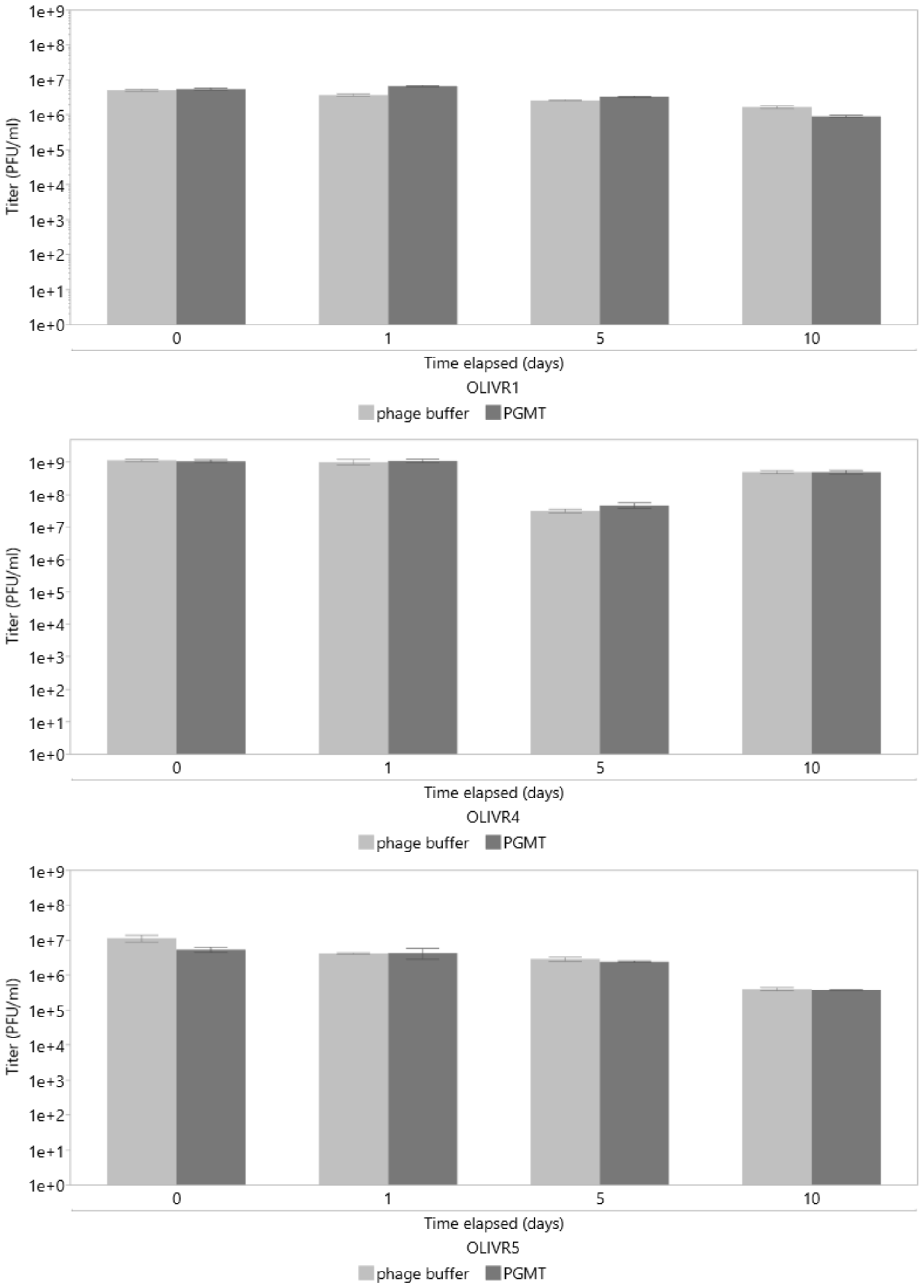

Figure A.8: ***Stability of the OLIVR phages in plant growth medium for tomato (PGMT)****. All phages remained stable over a period of ten days. Since all differences between the phages incubated in phage buffer and PGMT were well below 0.1 log units, the differences were considered to be biologically insignificant and no statistical test was performed.*

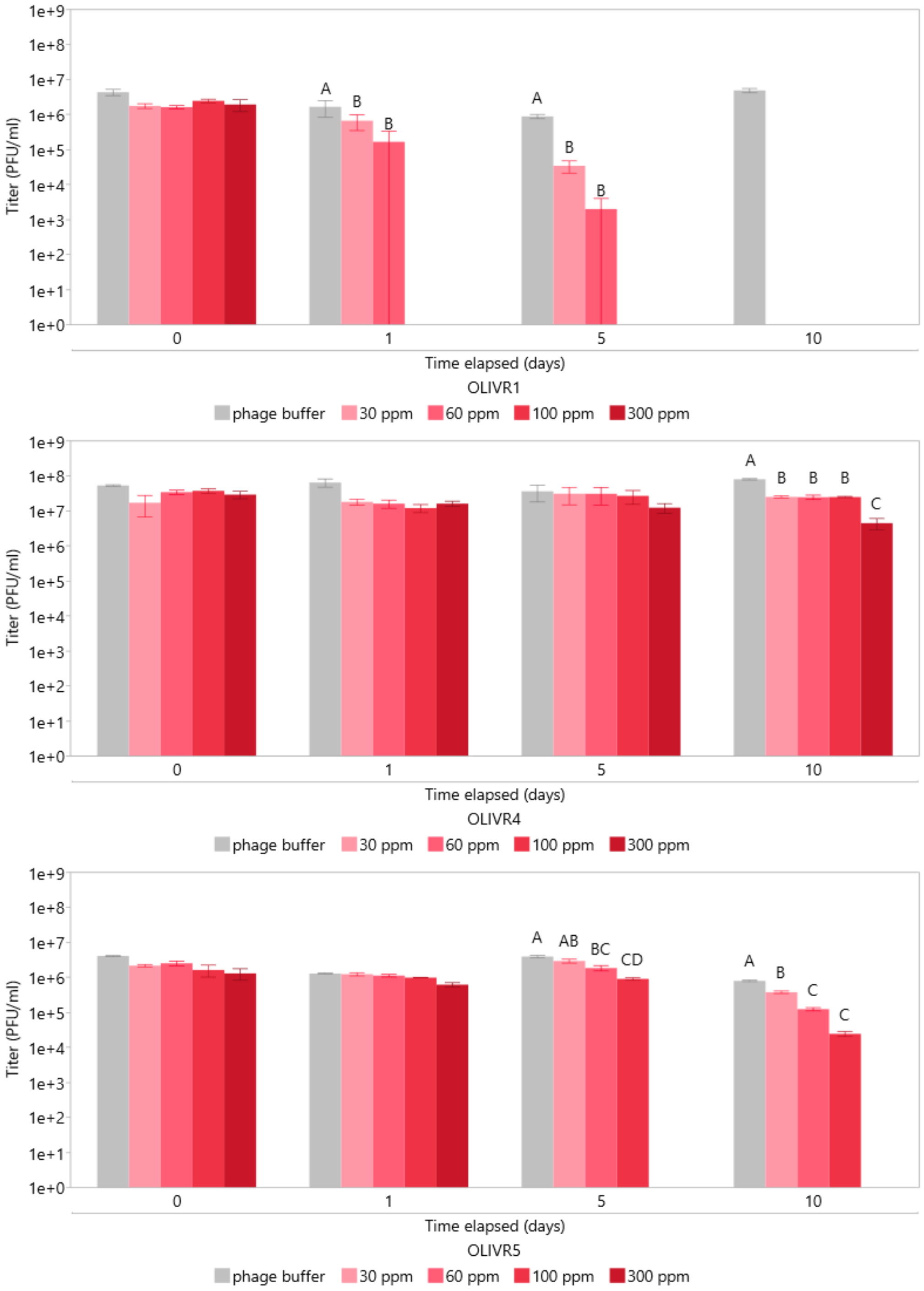

*Figure A.9: **Stability of the OLIVR phages in different concentrations of hydrogen peroxide**. The least stable phage was OLIVR1, which was totally inactivated in concentrations of 100ppm and higher within a day and steadily decreased in titer in concentrations of 30 and 60 ppm. OLIVR4 on the other hand remained stable in all concentrations for five days. After ten days though, a significant decrease was observed compared to the negative control. OLIVR5 remained stable in all concentration of hydrogen peroxide for one day. After five days, had significantly decreased in 100ppm and 300ppm, in which all phages were inactivated. In 30 and 60ppm, a significant decrease in phage concentration was observed only after ten days.*

Phage titers during the water disinfection assay

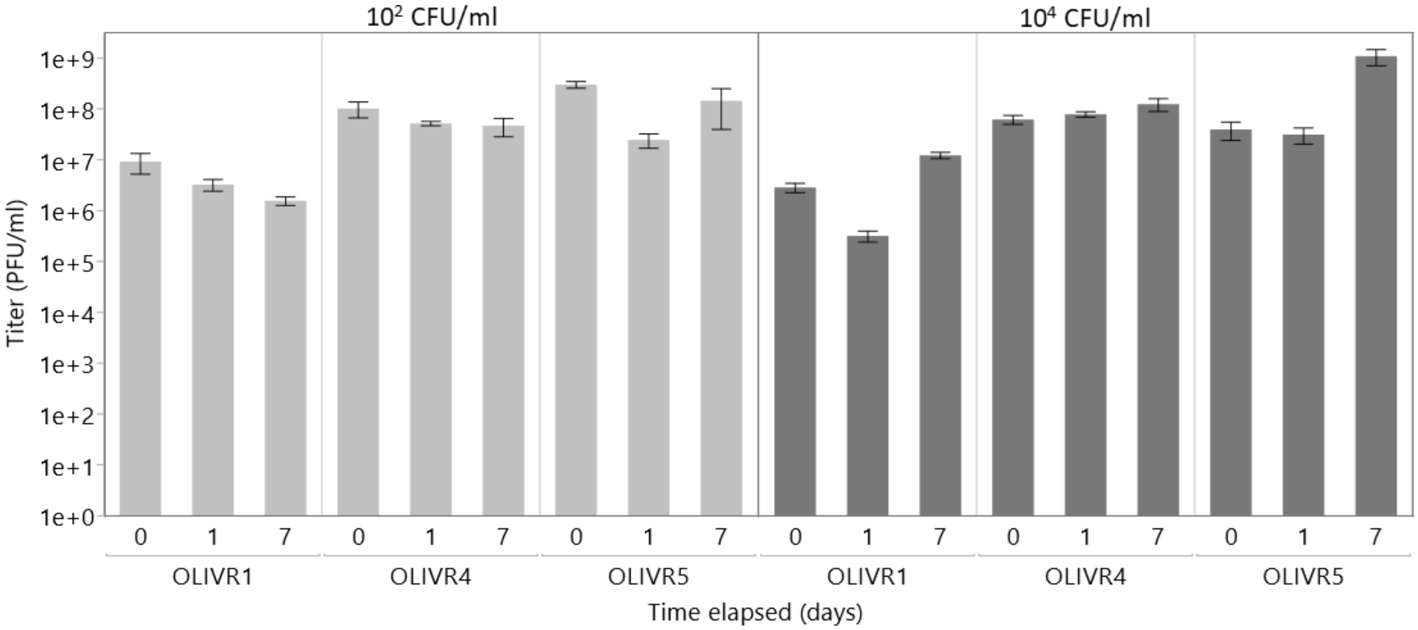

Figure A.10: ***The phage titer during the course of the water disinfection assay.*** *During the period of seven days, the presence of the phages was determined using a spot assay. As expected, the phage titer remained high during the entire experiment.*

Adsorption tests on the resistant isolates from the water disinfection assay and the receptor analysis

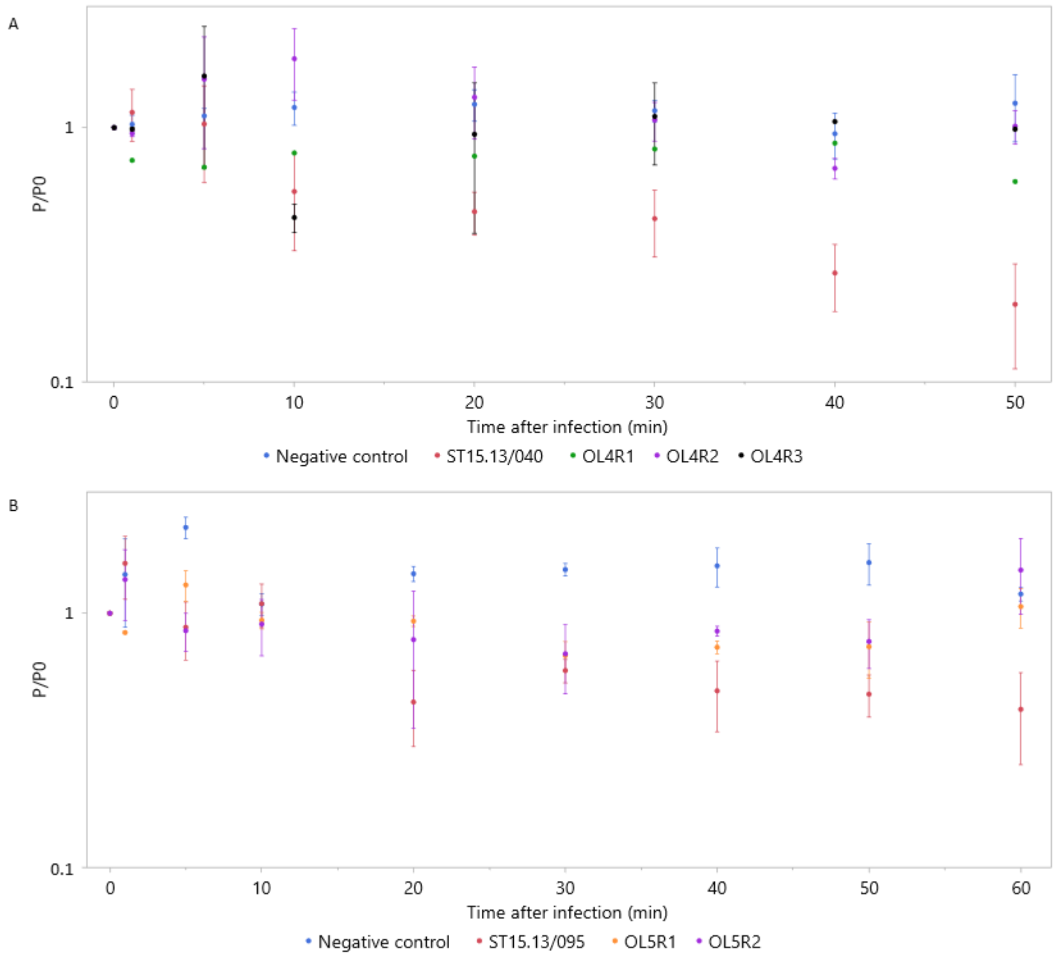

Figure A.11: ***Adsorption tests of the resistant strains obtained from the water disinfection assay****. (A) adsorption tests of OL4R1, OL4R2 and OL4R3. No decrease in free phage particles is observed in time. Hence there is no adsorption and resistance is a result of receptor modification. (B) adsorption tests of OL5R1 and OL5R2. No decrease in free phage particles is observed in time. Hence there is no adsorption and resistance is a result of receptor modification.*

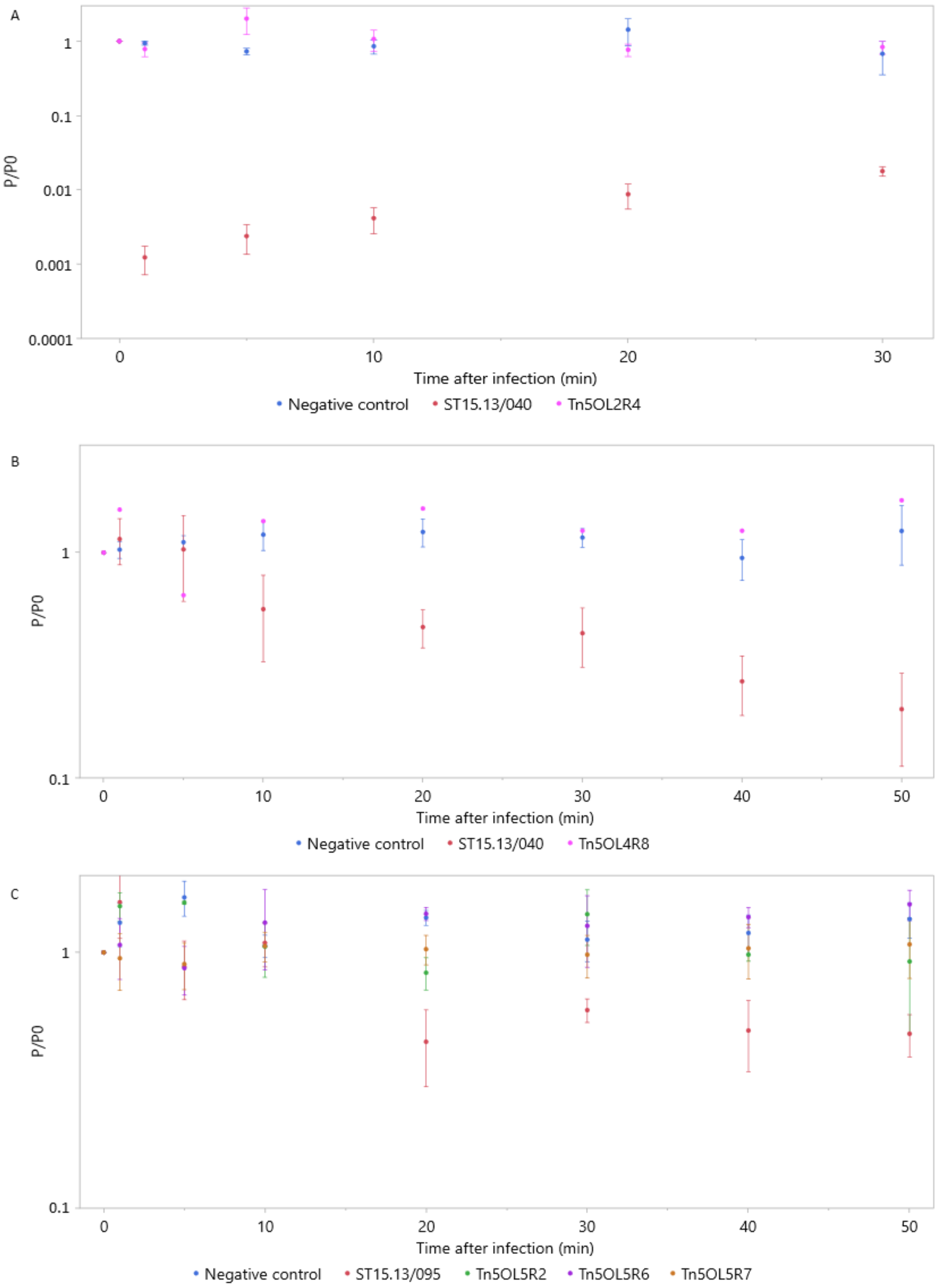

Figure A.12: ***Adsorption tests of the phage resistant mutants obtained during the transposon mutagenesis****. (A) adsorption tests with Tn5OL1R4 show no decrease in free phage concentration over time. Hence there is no adsorption. (B) adsorption tests with Tn5OL4R8 show no decrease in free phage concentration over time. Hence there is no adsorption. (C). Adsorption tests with Tn5OL5R2, Tn5OL5R6 and Tn5OL5R7 show no decrease in free phage concentration over time. Hence there is no adsorption.*

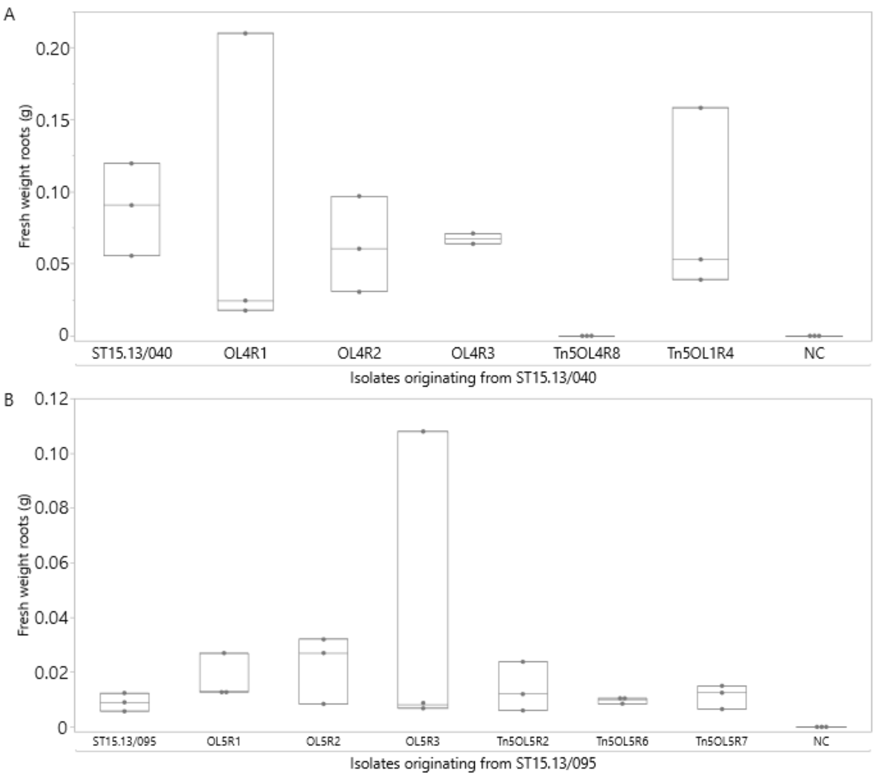

Figure A. 13: ***Bean virulence assays with ancestral strains and resistant isolates****. Bean assays showed that ancestral Agrobacterium, the resistant mutants from the water disinfection assay and the resistant mutants with transposon insertions all retained their ability to cause disease in this system. The only exception was Tn5OL4R8, which could no longer cause crazy roots.*
